# Supplementary figures and images for: Nanog safeguards early embryogenesis against global activation of maternal β-catenin activity by interfering with TCF factors
Source: PLoS Biol. 2020 Jul 23;18(7):e3000561. doi: 10.1371/journal.pbio.3000561 (PMC7402524; doi:10.1371/journal.pbio.3000561)

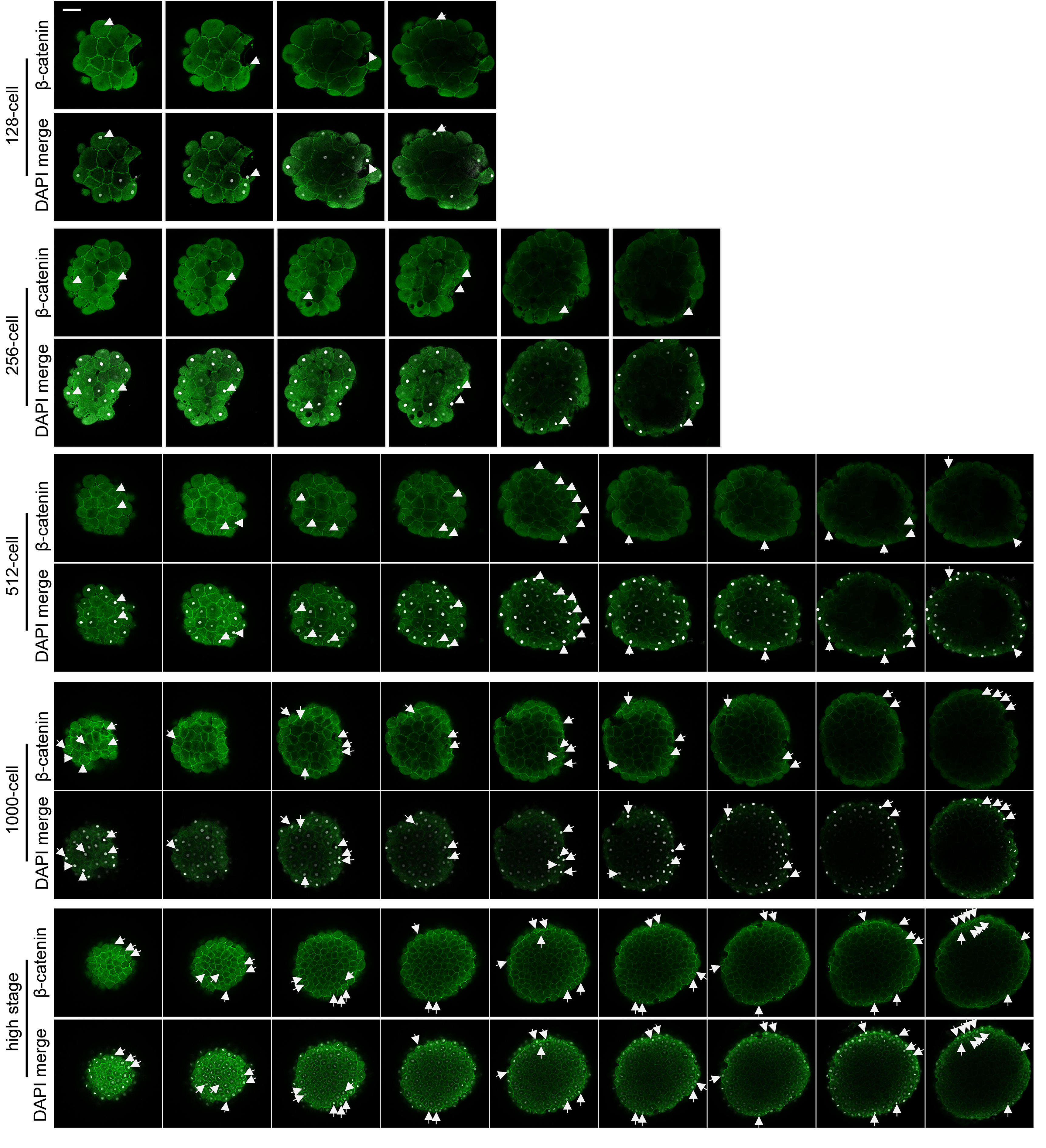

Supplement: S1 Fig — Nuclear β-catenin distribution in zebrafish blastodermal cells was detected by whole-mount Immunofluorescence from 128-cell stage to high stage. Embryos were mounted, and the signal was detected at animal view. Nuclei were co-stained with DAPI. At least 15 embryos were detected in each stage. Arrow heads indicate the nuclear accumulation of β-catenin. Scale bar, 50 μm. (TIF) [file pbio.3000561.s001.tif]

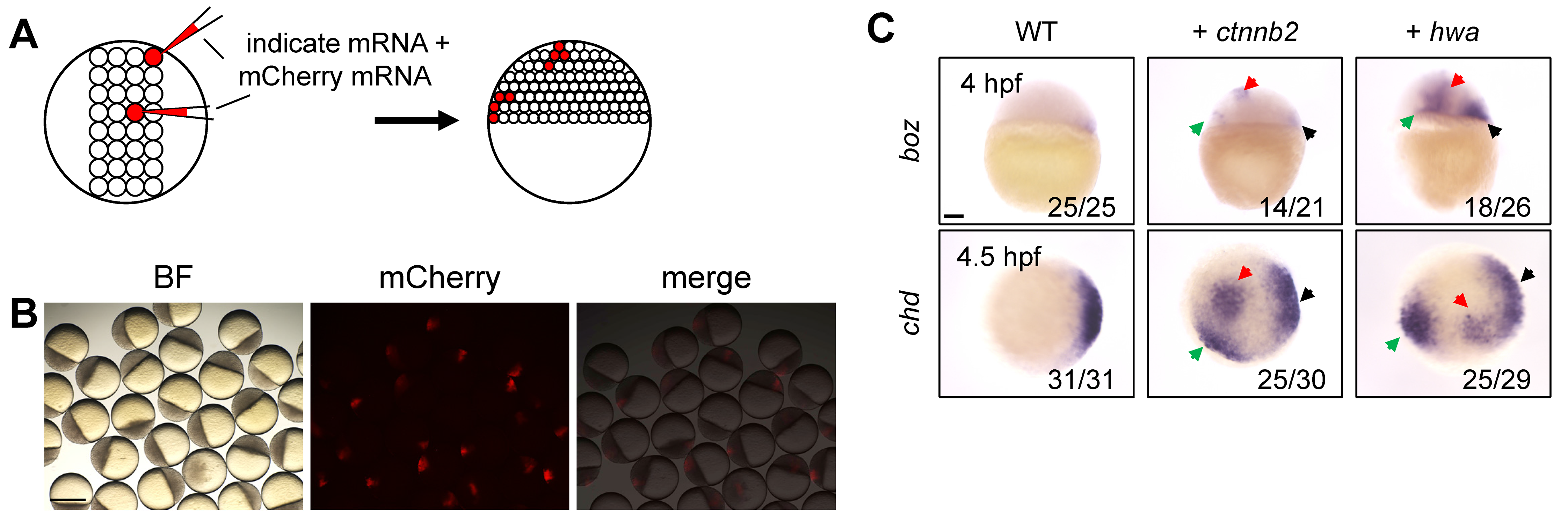

Supplement: S2 Fig — (A) A diagram of localized injection into 2 cells at 32-cell stage. One injected cell is on the margin, and the other injected cell is located at the center of blastula. (B) ctnnb2 or hwa mRNA was co-injected with mCherry mRNA and located injected mCherry fluorescence were observed at 4 hpf. Scale bar, 500 μm. (C) WISH analysis showing the ectopic expression of boz and chd induced by ctnnb2 and hwa mRNA. Embryos with mCherry fluorescence were collected and examined by WISH. boz was detected at 4 hpf, and chd was detected at 4.5 hpf. The numbers below the WISH pictures are the number of embryos showing representative phenotype/total number of embryos. Scale bar, 100 μm. hpf, hours post fertilization; WISH, whole-mount in situ hybridization; WT, wild type. (TIF) [file pbio.3000561.s002.tif]

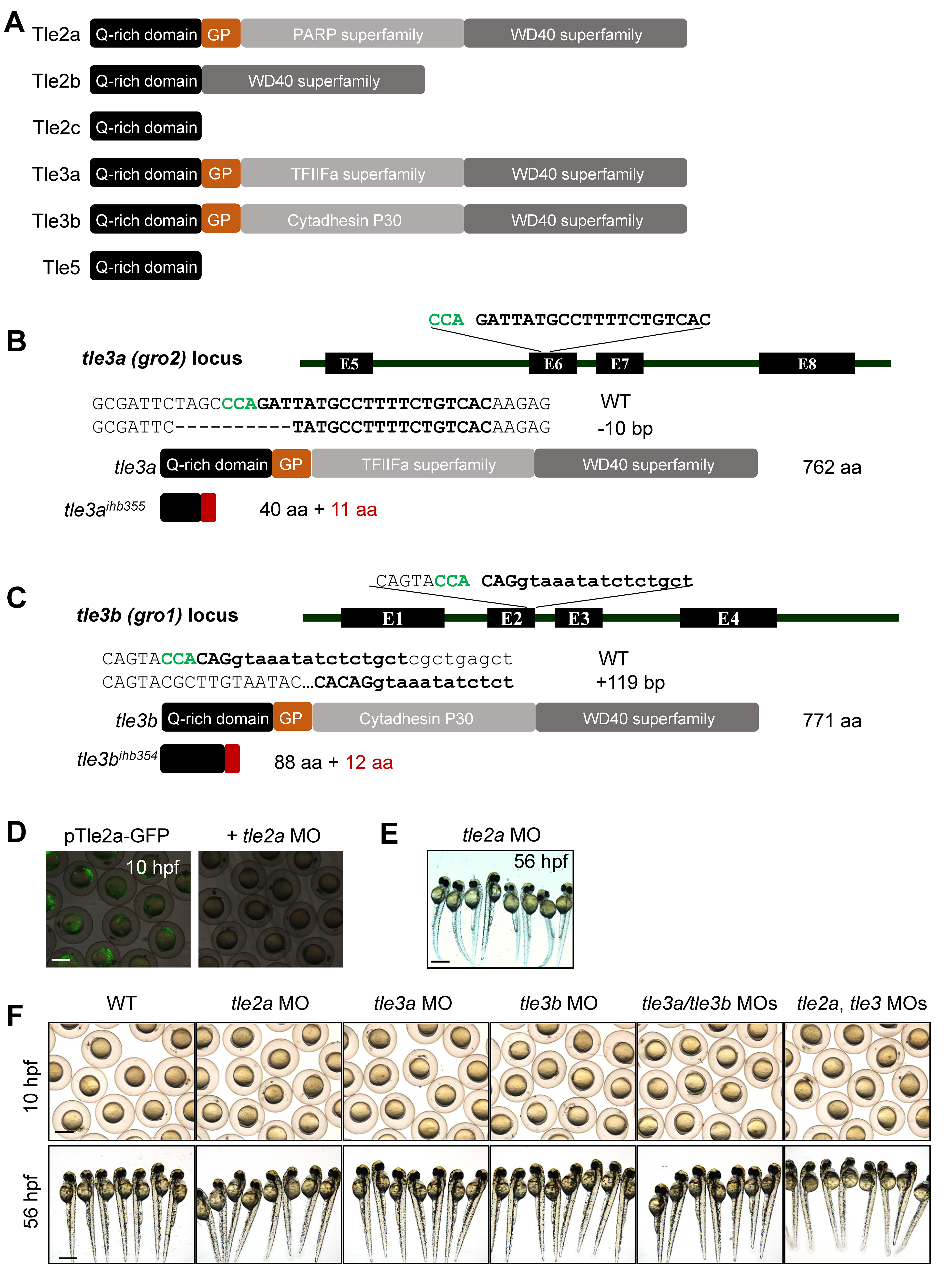

Supplement: S3 Fig — (A) Functional domain analysis of zebrafish Tle2a, Tle2b, Tle2c, Tle3a, Tle3b, and Tle5. (B) The CRISPR/Cas9 target of tle3a is located within exon 6, and a 10-bp deletion mutant (tle3aihb355) was obtained. (C) The CRISPR/Cas9 target of tle3b is located at the splicing site of exon 2 and intron 2, and a 119-bp insertion mutant (tle3bihb354) was obtained. Target sequence is in bold, and the PAM sequence is in green. (D) Validation of tle2a MO effectiveness. tle2a MO target sequence was fused with GFP to result in pTle2a-GFP construct. pTle2a-GFP construct or pTle2a-GFP combination with tle2a MO was injected at one-cell stage. Fluorescence was observed at 10 hpf. Scale bar, 500 μm. (E) Knockdown of tle2a at 2 ng/embryo did not affect the early development of zebrafish. Scale bar, 500 μm. (F) Knockdown of tle2a (2 ng/embryo), tle3a (2 ng/embryo), and tle3b (2 ng/embryo), respectively, or in combination, does not lead to early embryonic development defect at 10 hpf and 56 hpf. MO or combined MOs was injected at one-cell stage, and at least 50 embryos were injected and observed. Scale bar, 500 μm. bp, base pair; GFP, green fluorescent protein; hpf, hours post fertilization; MO, morpholino; PAM, protospacer adjacent motif; WT, wild type. (TIF) [file pbio.3000561.s003.tif]

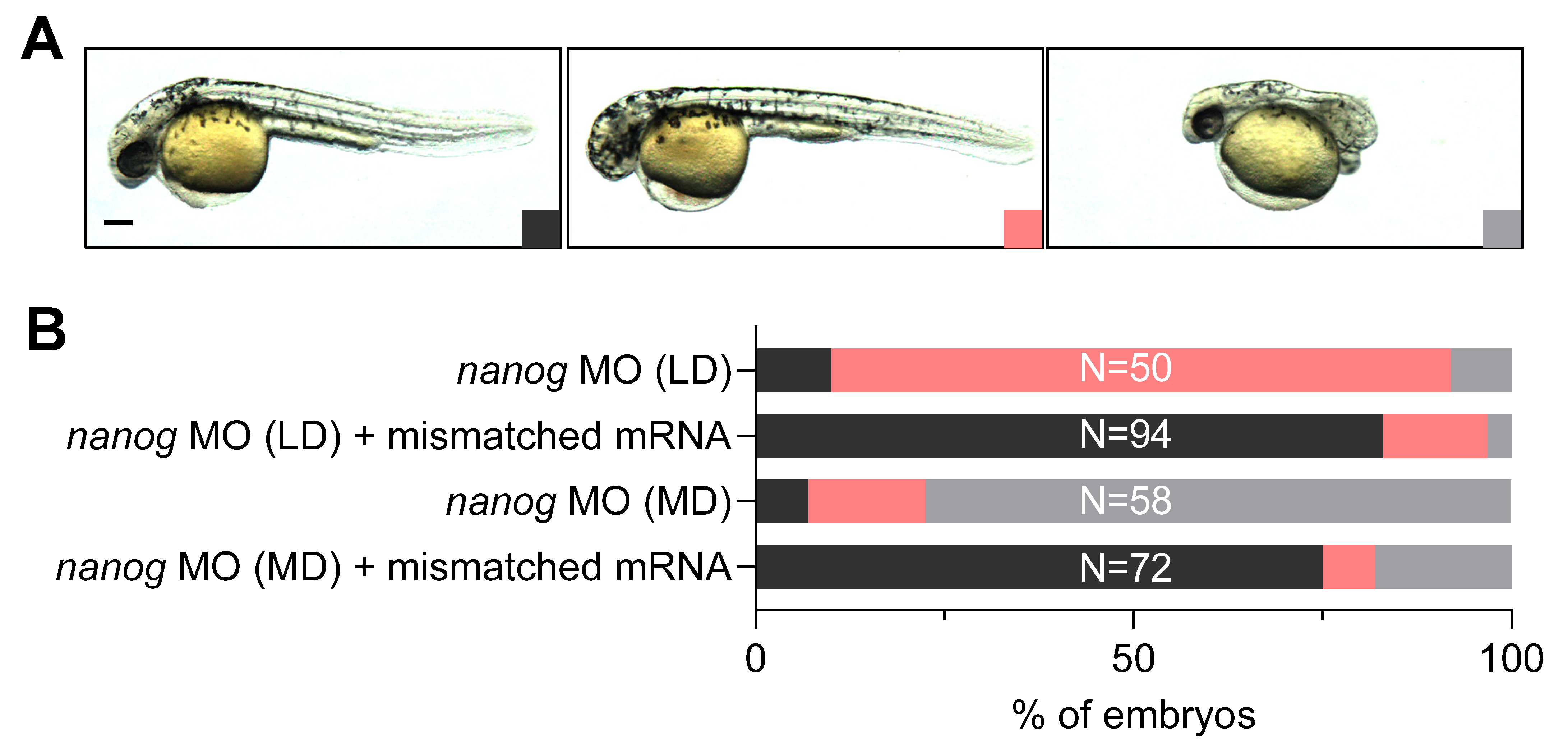

Supplement: S4 Fig — (A) Three classes of phenotypes—WT-like, posterization, and dorsalization—were characterized in nanog morphants at 36 hpf. Scale bar, 100 μm. (B) Overexpression of nanog mismatched mRNA (nanog MO targeted site is mutated) rescued the posterization, and dorsalization defects of nanog morphants. N represents analyzed embryo number. The underlying data in this figure can be found in S1 Data. hpf, hours post fertilization; MO, morpholino; WT, wild type. (TIF) [file pbio.3000561.s004.tif]

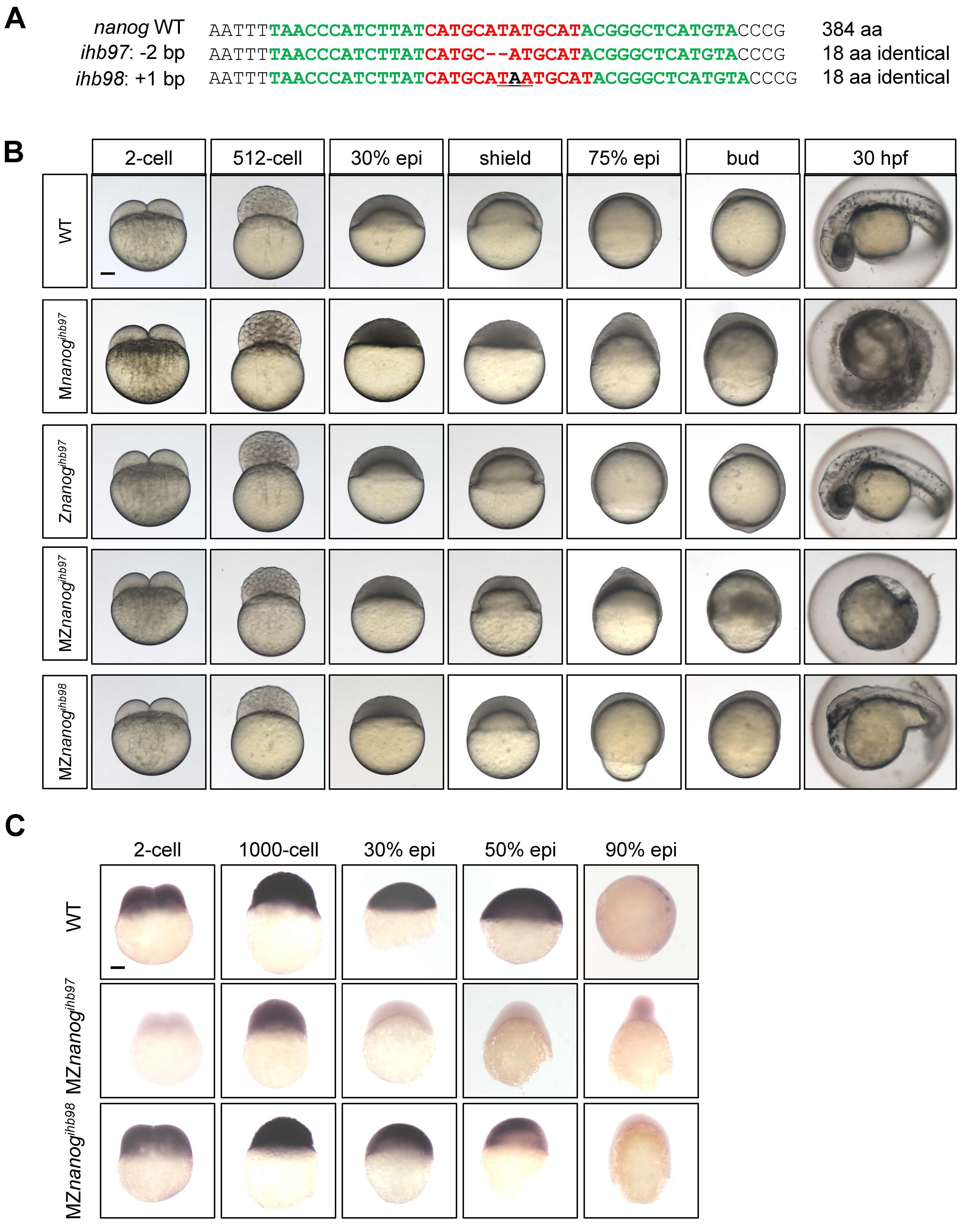

Supplement: S5 Fig — (A) Generation of 2 nanog mutant alleles by TALEN. TALEN left and right arms are marked in green, and the target sequence is in red. Two different mutant lines were obtained: 2-bp deletion line (nanogihb97) and 1-bp insertion line (nanogihb98). The 2-bp deletion leads to frame-shift of Nanog protein, and the 1-bp insertion results in premature termination of Nanog protein at the mutation site. (B) Phenotype characterization of Mnanogihb97, Znanogihb97, and 2 different mutants, MZnanogihb97 and MZnanogihb98. Both of Mnanogihb97 and two types of maternal -zygotic nanog mutants, MZnanogihb97, MZnanogihb98 show slow development and abnormal cell movement, then die within 24 hpf. However, Znanogihb97 mutant shows normal development and reproduction, the same as WT. Scale bar, 100 μm. (C) Maternal nanog expression disappeared and small amount of zygotic nanog was detected in MZnanogihb97. Both low expression of maternal and zygotic nanog were detected in MZnanogihb98. Scale bar, 100 μm. bp, base pair; hpf, hours post fertilization; Mnanog, maternal mutant of nanog; MZnanog, maternal -zygotic mutant of nanog; TALEN, transcription activator-like effector nuclease; WT, wild type. (TIF) [file pbio.3000561.s005.tif]

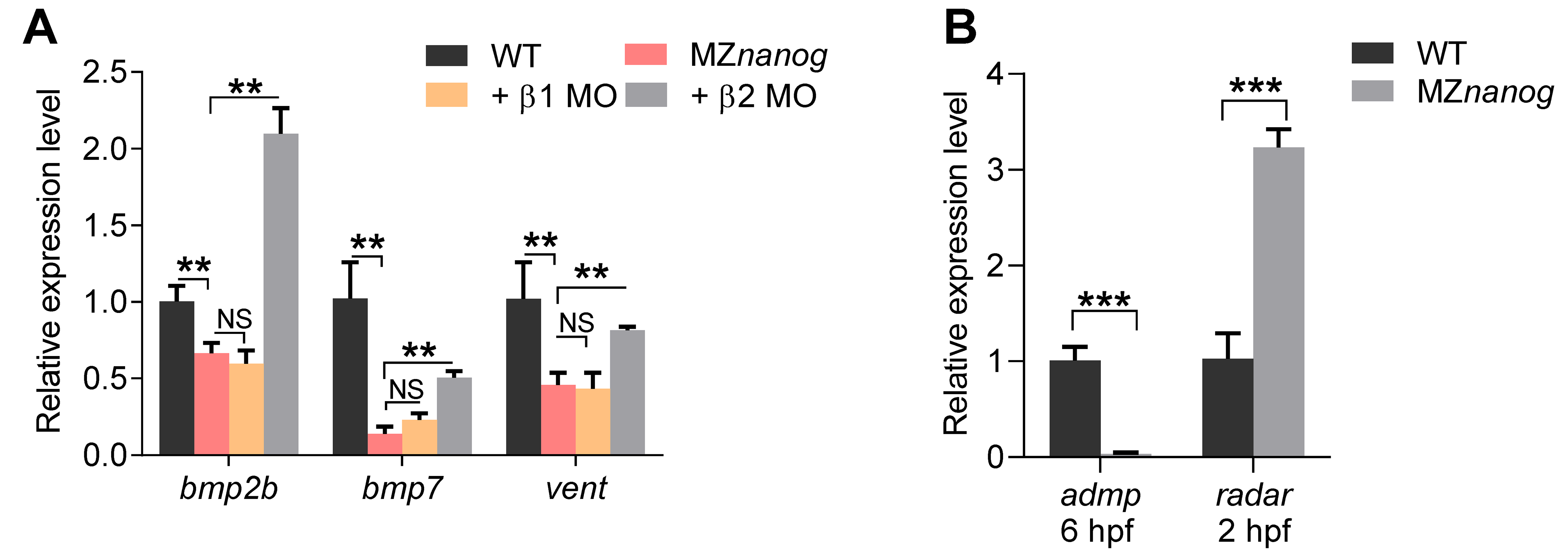

Supplement: S6 Fig — (A) Relative mRNA level of bmp2b, bmp7, and BMP target vent were reduced in MZnanog examined by RT-qPCR analysis. Depletion of ctnnb2 could restore the abnormal expression of bmp2b, bmp7, and vent, whereas knockdown of ctnnb1 could not. bmp2b, bmp7, and vent was detected at 6 hpf. β1 MO, ctnnb1 MO; β2 MO, ctnnb2 MO. Error bars, mean ± SD, **P < 0.01; NS means no significant difference. (B) Relative mRNA level of admp was significantly decreased in MZnanog at 6 hpf, and radar was significantly up-regulated at 2 hpf by RT-qPCR analysis. Error bars, mean ± SD, ***P < 0.001. The P values in this figure were calculated by Student t test. The underlying data in this figure can be found in S1 Data. hpf, hours post fertilization; MO, morpholino; MZnanog, maternal zygotic mutant of nanog; RT-qPCR, reverse-transcription quantitative PCR; WT, wild type. (TIF) [file pbio.3000561.s006.tif]

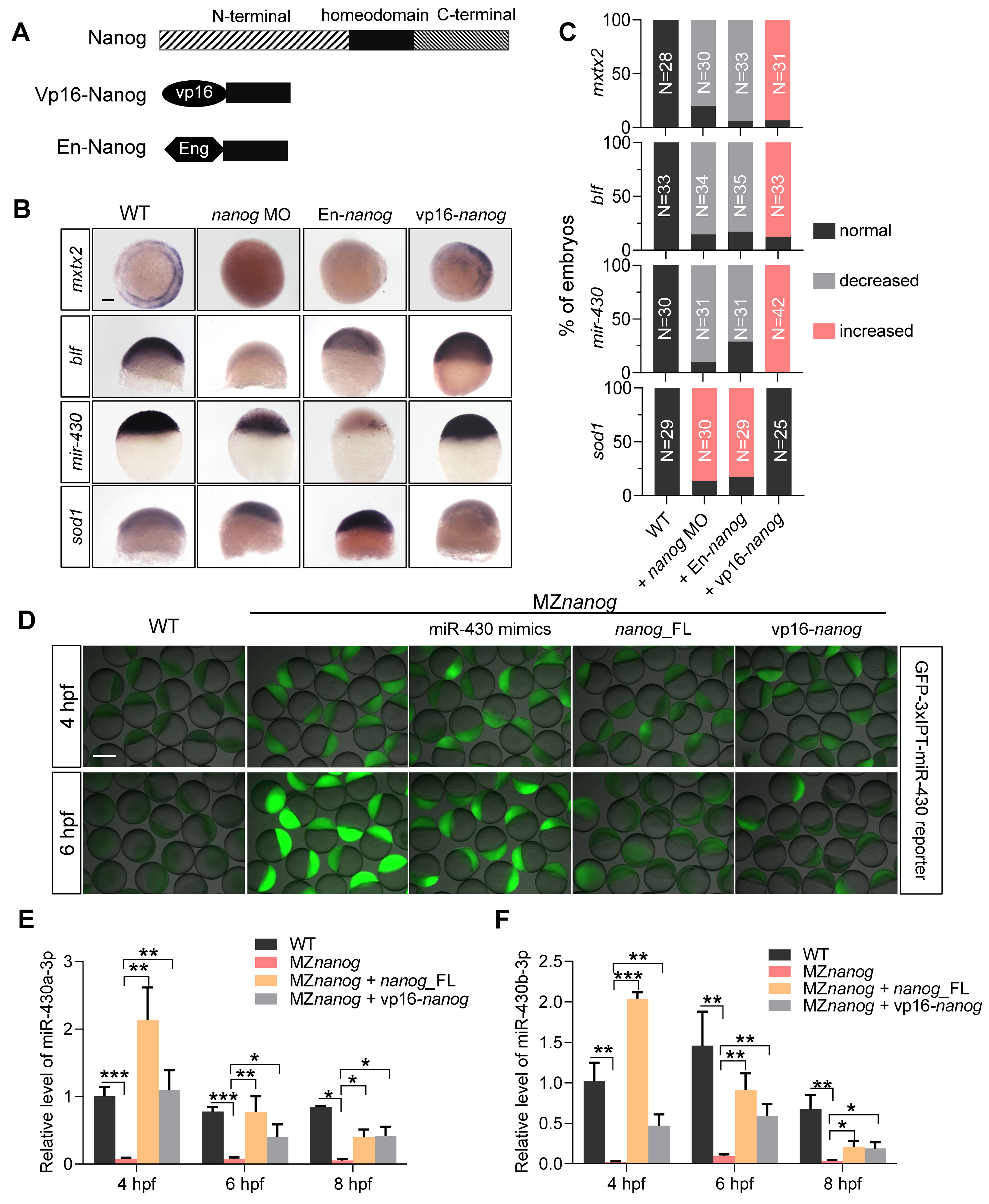

Supplement: S7 Fig — (A) The diagram of Vp16-Nanog (vp16-Nanog homeodomain) and En-Nanog (Enrgrailed2-Nanog homeodomain). The transcription activator VP16 or repressor Enrgrailed2 was fusion with Nanog homeodomain to result in Vp16-Nanog or En-Nanog. (B) WISH analysis showing the expression of mesendoderm marker, mxtx2, strictly zygotic gene, blf, and microRNA-430 precursor (mir-430), and miR-430 target, sod1 in embryos of WT, WT injected with nanog MO, En-nanog or vp16-nanog. Expression of mxtx2, blf, and mir-430 was reduced, even absent, in nanog morphants and En-Nanog injected embryos but enhanced in vp16-nanog overexpressed embryos. The expression of miR-430 target gene, sod1, failed to be cleared in nanog morphants and En-Nanog injected embryos at shield stage. Scale bar, 100 μm. (C) Statistical analysis of embryos in panel B. N represents analyzed embryo number. (D) The fluoresce intensity of GFP-3xIPT-miR-430 reporter, which carries a target sequence of miR-430, is negative correlated with the expression of miR-430. The intensity of GFP was higher in MZnanog than WT at 4 hpf and 6 hpf, indicating deletion of nanog resulted in inactivation of miR-430 expression. Meanwhile, overexpression of miR-430 mimics, nanog, or vp16-nanog in MZnanog restored the high expression of GFP-3xIPT-miR-430 reporter. Scale bar, 500 μm. (E and F) Relative expression level of miR-430a (E) and miR-430b (F) were inactivated in MZnanog examined by stem-loop PCR; overexpression of nanog_FL or vp16-nanog can fully restore the expression failure of miR-430a and miR-430b at 4 hpf, 6 hpf, and 8 hpf. Error bars, mean ± SD, *P < 0.05, **P < 0.01, ***P < 0.001. The P values in this figure were calculated by Student t test. The underlying data in this figure can be found in S1 Data. hpf, hours post fertilization; MZnanog, maternal -zygotic mutant of nanog; MZT, maternal zygotic transition; RT-qPCR, reverse-transcription quantitative PCR; WISH, whole-mount in situ hybridization; WT, wild type. (TIF) [file pbio.3000561.s007.tif]

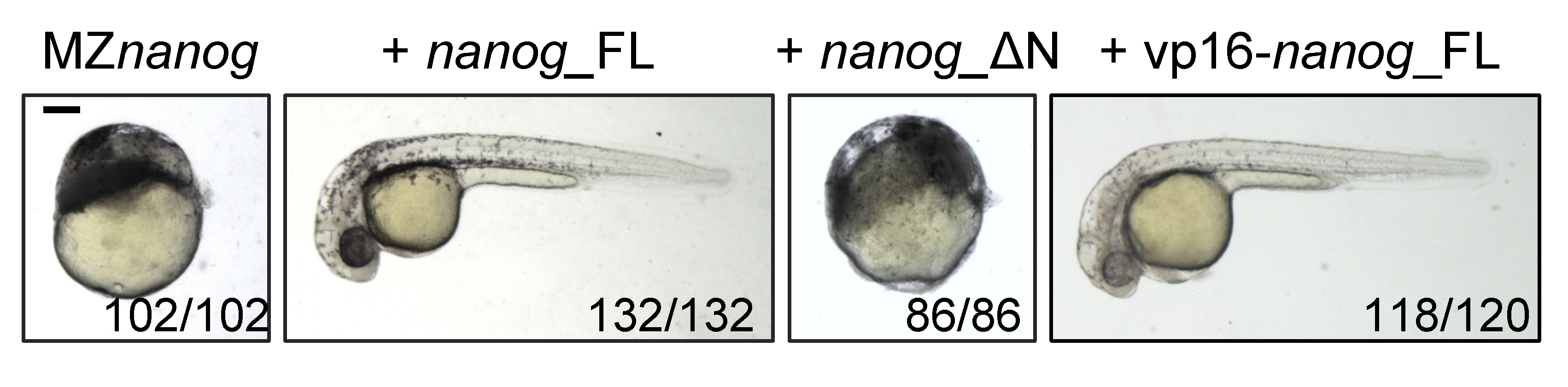

Supplement: S8 Fig — Overexpression of nanog_FL and vp16-nanog_FL could fully rescue the developmental defect of MZnanog, and N-terminal truncated nanog (nanog_ΔN) could not. The numbers below the embryo pictures are the number of embryos showing representative phenotype/total number of embryos. Scale bar, 100 μm. MZnanog, maternal zygotic mutant of nanog; nanog_FL, full length of Nanog; nanog_ΔN, N′ terminal truncated Nanog; vp16-nanog_FL, full length of Nanog fusion with Vp16. (TIF) [file pbio.3000561.s008.tif]

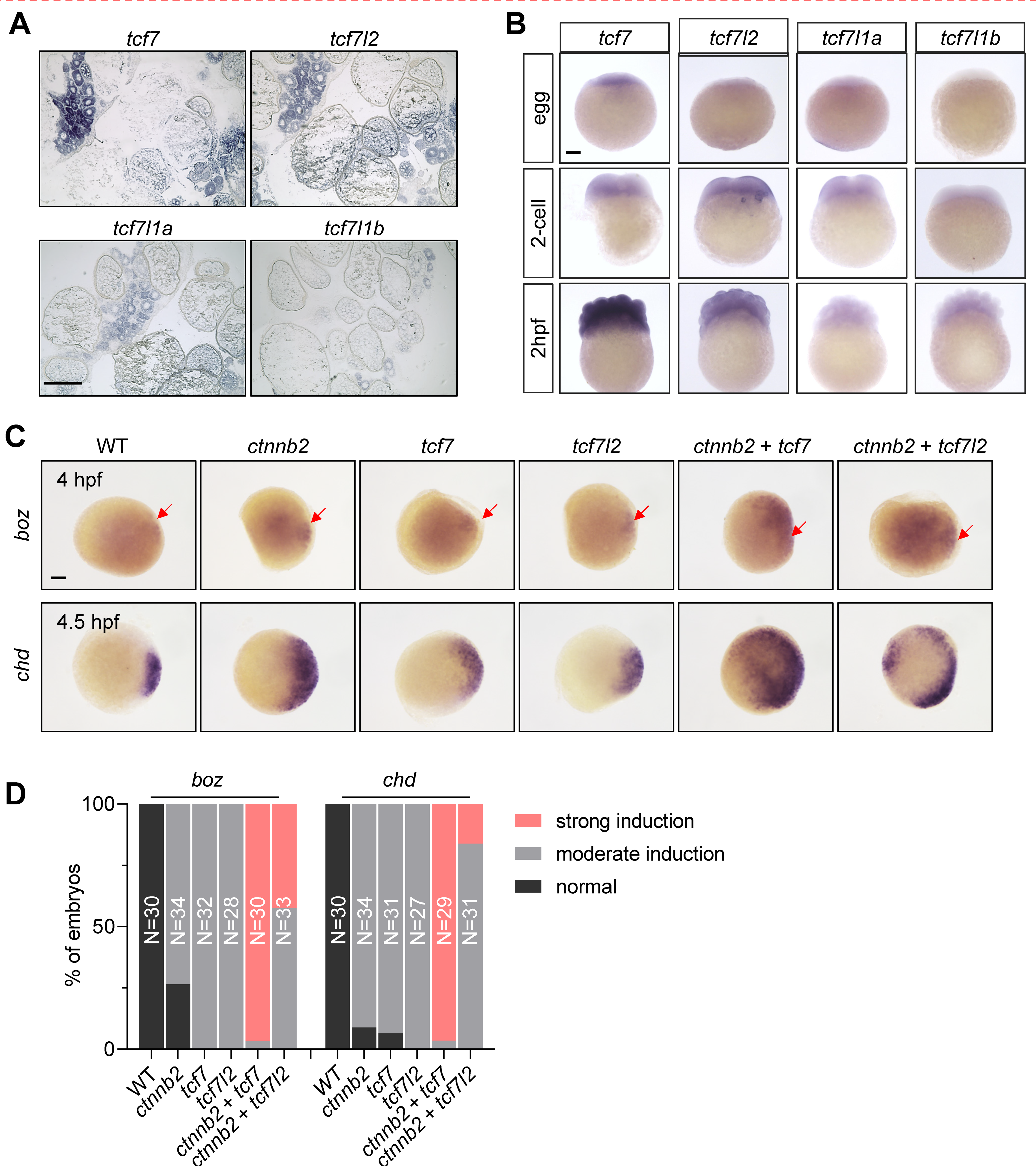

Supplement: S9 Fig — (A) Maternal expression of tcf7, tcf7l2, tcf7l1a, and tcf7l1b was detected by in situ hybridization on cryosections of ovaries. Scale bar, 100 μm. (B) WISH analysis showing tcf7, tcf7l2, tcf7l1a, and tcf7l1b were maternally deposited at unfertilized egg, 2-cell stage, and 2 hpf embryos. Scale bar, 100 μm. (C) Injection of low dose of ctnnb2 mRNA (200 pg/embryo), tcf7 mRNA, or tcf7l2 mRNA alone, or co-injection of low dose of ctnnb2 mRNA (200 pg/embryo) with tcf7, or tcf7l2 at 1-cell stage and detection the expression of boz and chd by WISH. Both individual injection and co-injection of TCF with ctnnb2 mRNA induced up-regulation of boz and chd, and Tcf7 coordination with Ctnnb2 showed more efficient induction of boz and chd than Tcf7l2 and Ctnnb2, suggesting Tcf7 could serve as a strong activator-type TCF in mediating maternal β-catenin activity. boz was detected at 4 hpf; chd was detected at 4.5 hpf. Scale bar, 100 μm. (D) Statistical analysis of the embryos in panel C. N represents analyzed embryo number. The underlying data in this figure can be found in S1 Data. hpf, hours post fertilization; TCF, T-cell factor; WISH, whole-mount in situ hybridization; WT, wild type. (TIF) [file pbio.3000561.s009.tif]

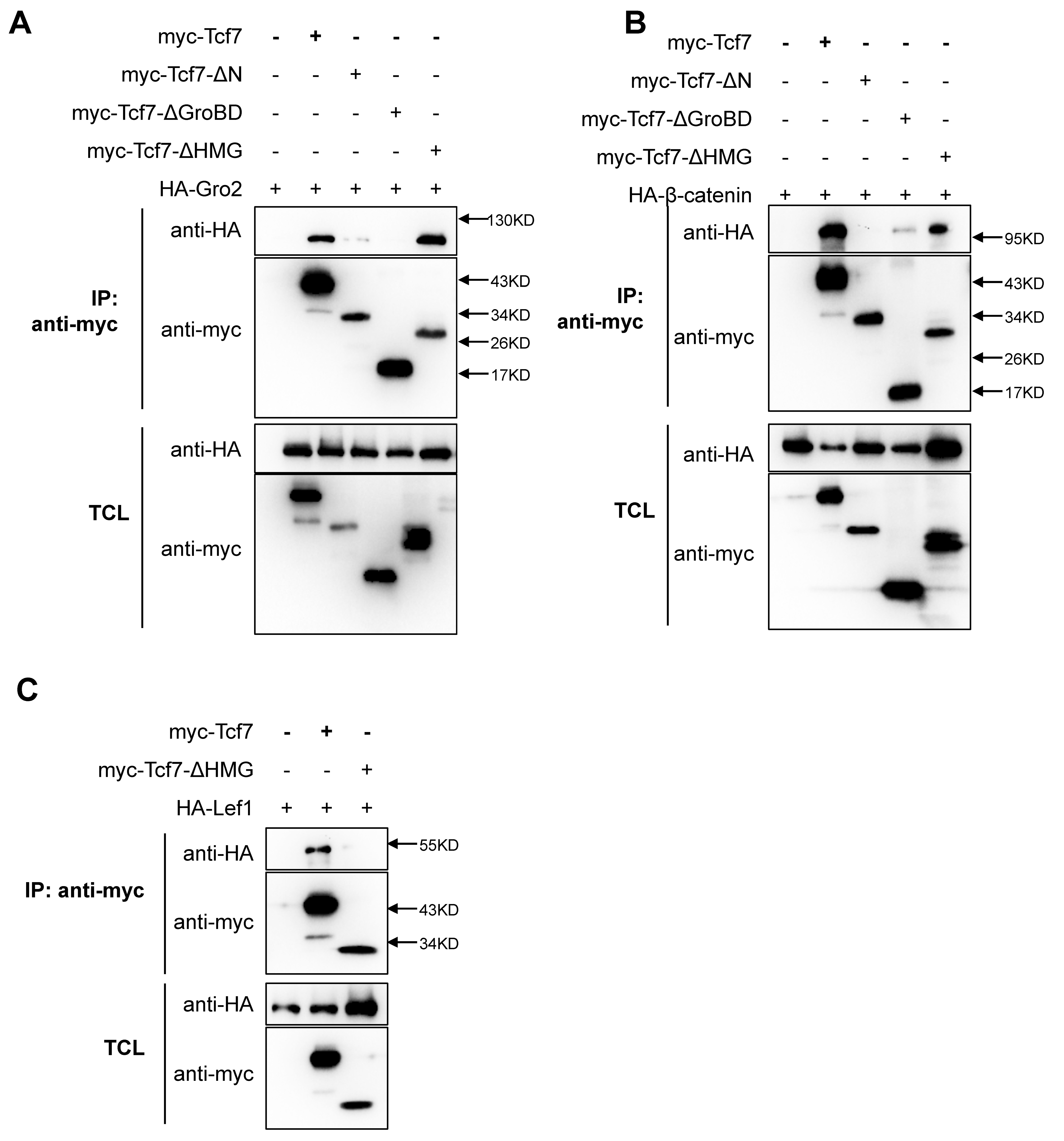

Supplement: S10 Fig — (A) Tcf7 interacts with Groucho/TLE through predicted GroBD. Different Myc-tagged Tcf7 were constructed and co-transfection with HA-Groucho2 in HEK293T cells. Deletion of predicted GroBD of Tcf7 disrupts its interaction with Groucho2, indicating that Groucho2 physically binds to the potential GroBD of Tcf7. (B) Tcf7 interacts with β-catenin through its N terminal. Deletion of the N terminal of Tcf7 disrupts its interaction with β-catenin, indicating that β-catenin physically interacts with the N terminal of Tcf7. (C) Tcf7 interacts with LEF1 through its HMG domain (C terminal). Deletion of HMG domain of Tcf7 disrupts its interaction with Lef1, indicating that Lef1 physically interacts with the HMG domain of Tcf7. GroBD, Groucho/TLE binding domain; HEK293T, human embryonic kidney 293T; HMG, high mobility group; TLE, transducin-like enhancer of split. (TIF) [file pbio.3000561.s010.tif]
