# Supplementary material for: Nanog safeguards early embryogenesis against global activation of maternal β-catenin activity by interfering with TCF factors
Source: PLoS Biol. 2020 Jul 23;18(7):e3000561. doi: 10.1371/journal.pbio.3000561 (PMC7402524; doi:10.1371/journal.pbio.3000561)
Supplement: S1 Raw Images — (PDF) [file pbio.3000561.s011.pdf]

Uncropped blots for Figure 2C

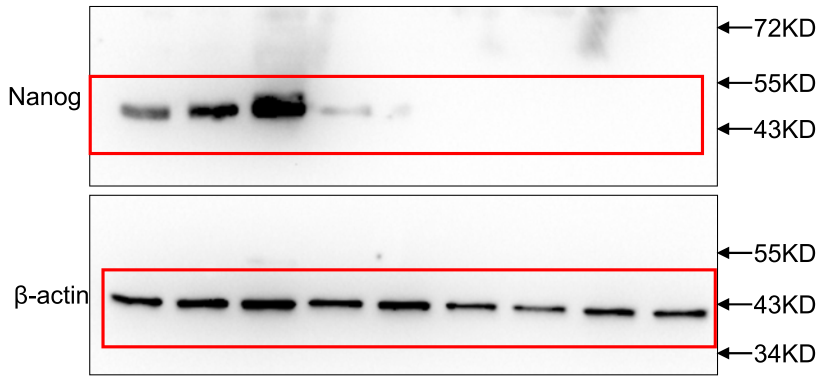

Uncropped blots for Figure 5D

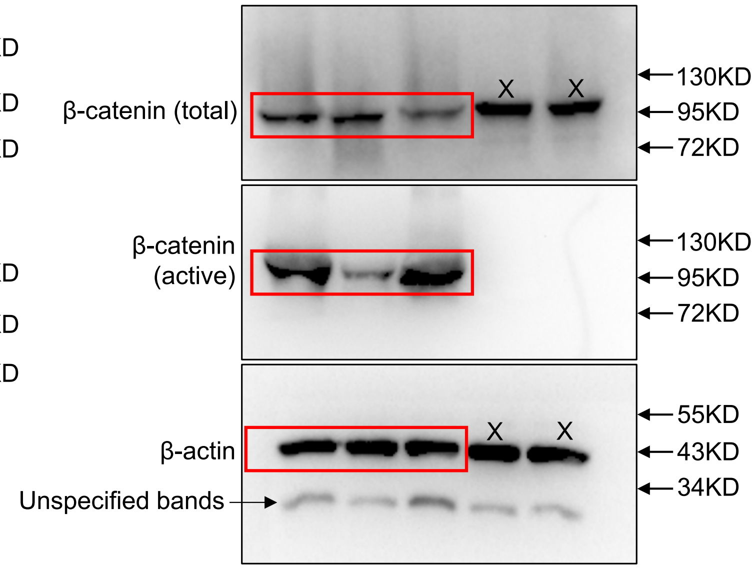

Uncropped blots for Figure 4A

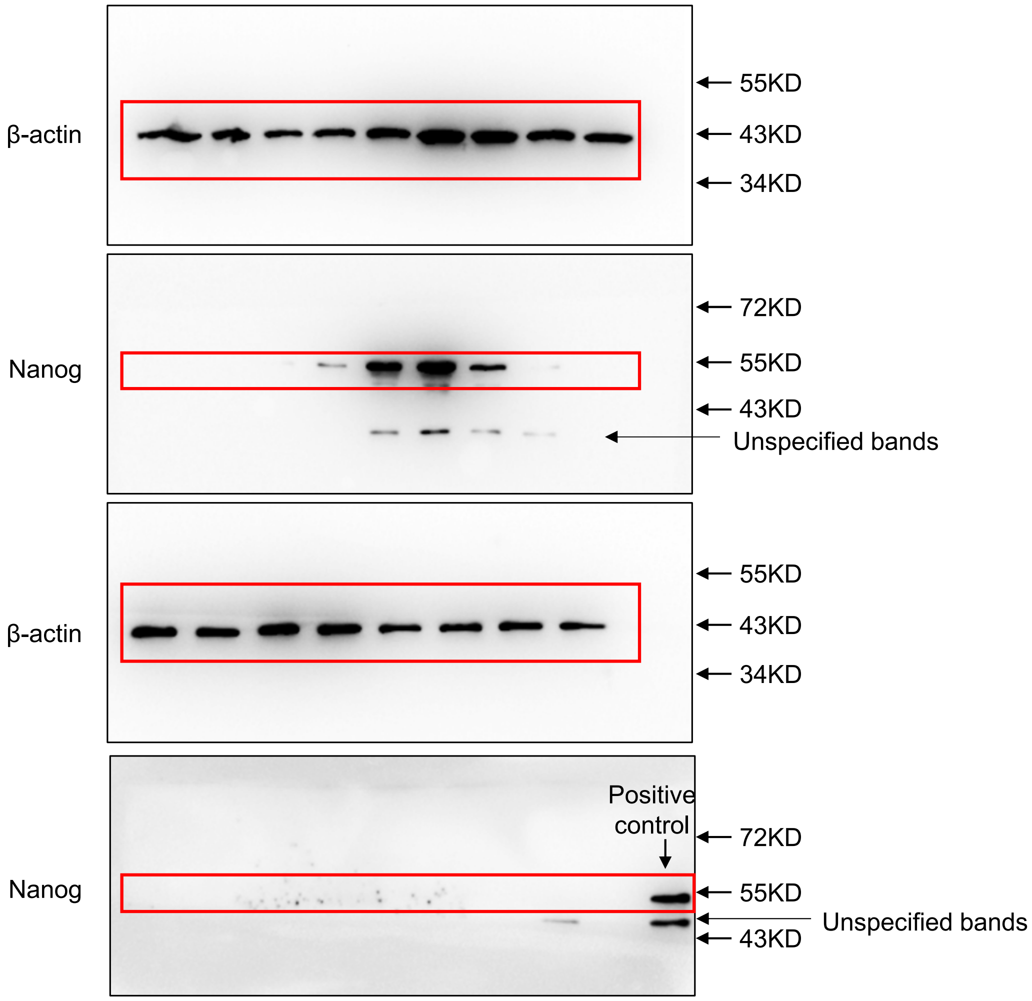

Images were acquired by ChemicDoc MP imaging system (BioRad, USA). Approximate molecular weight ladder was indicated. PVDF Membranes were cut for immunoblotting of more than one protein.

Uncropped blots for Figure 7A

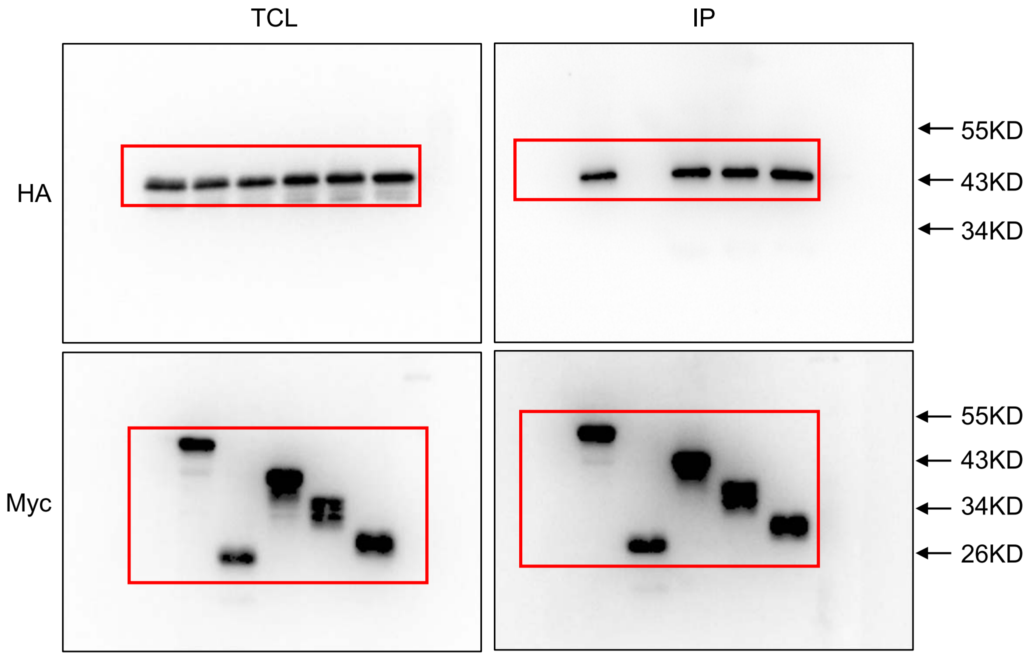

Uncropped blots for Figure 7B

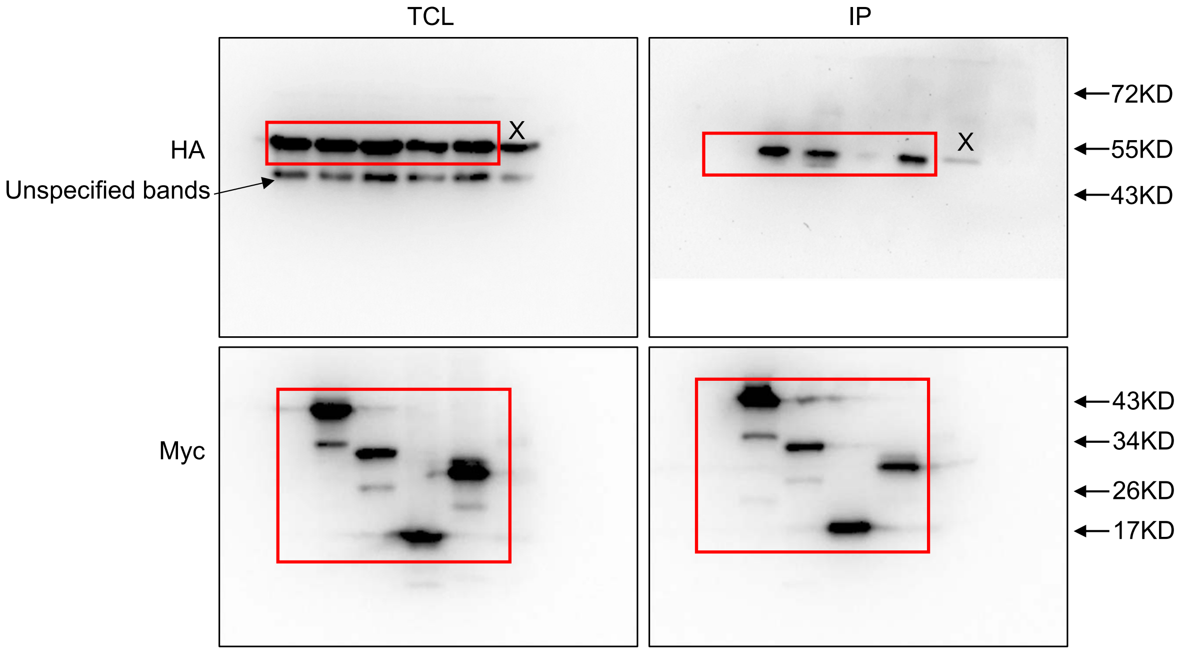

Images were acquired by ChemicDoc MP imaging system (BioRad, USA). Approximate molecular weight ladder was indicated. PVDF Membranes were cut for immunoblotting of more than one protein.

Uncropped blots for Figure 7C

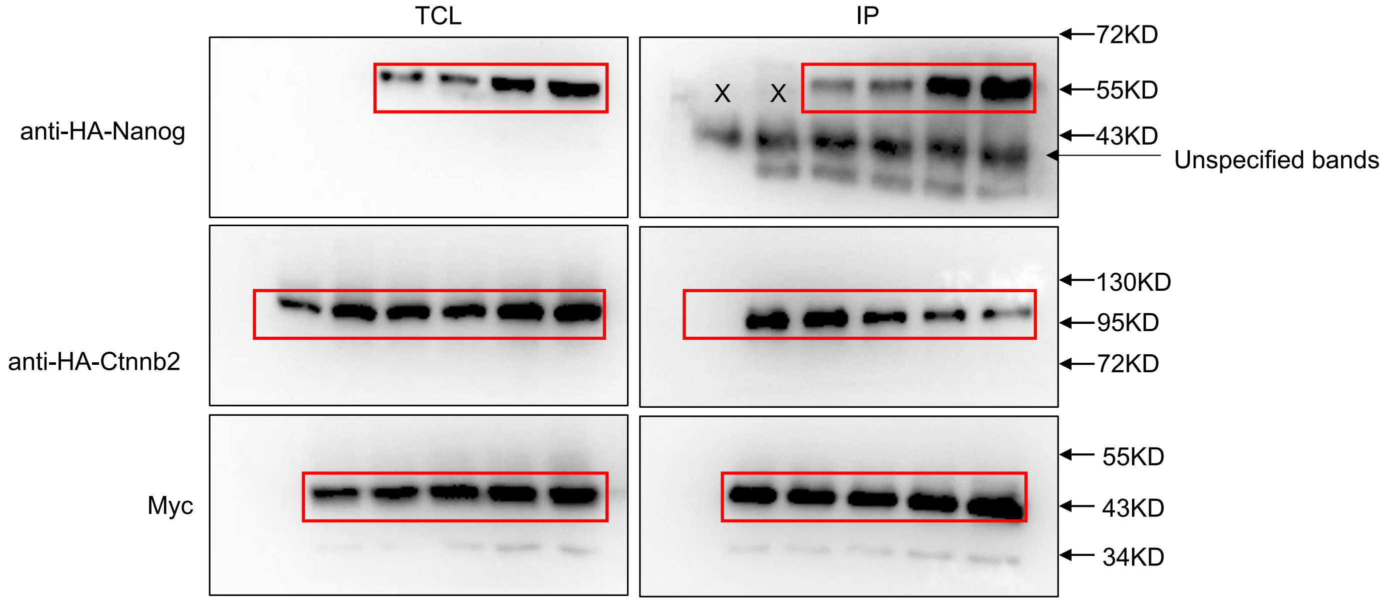

Uncropped blots for Figure 7D

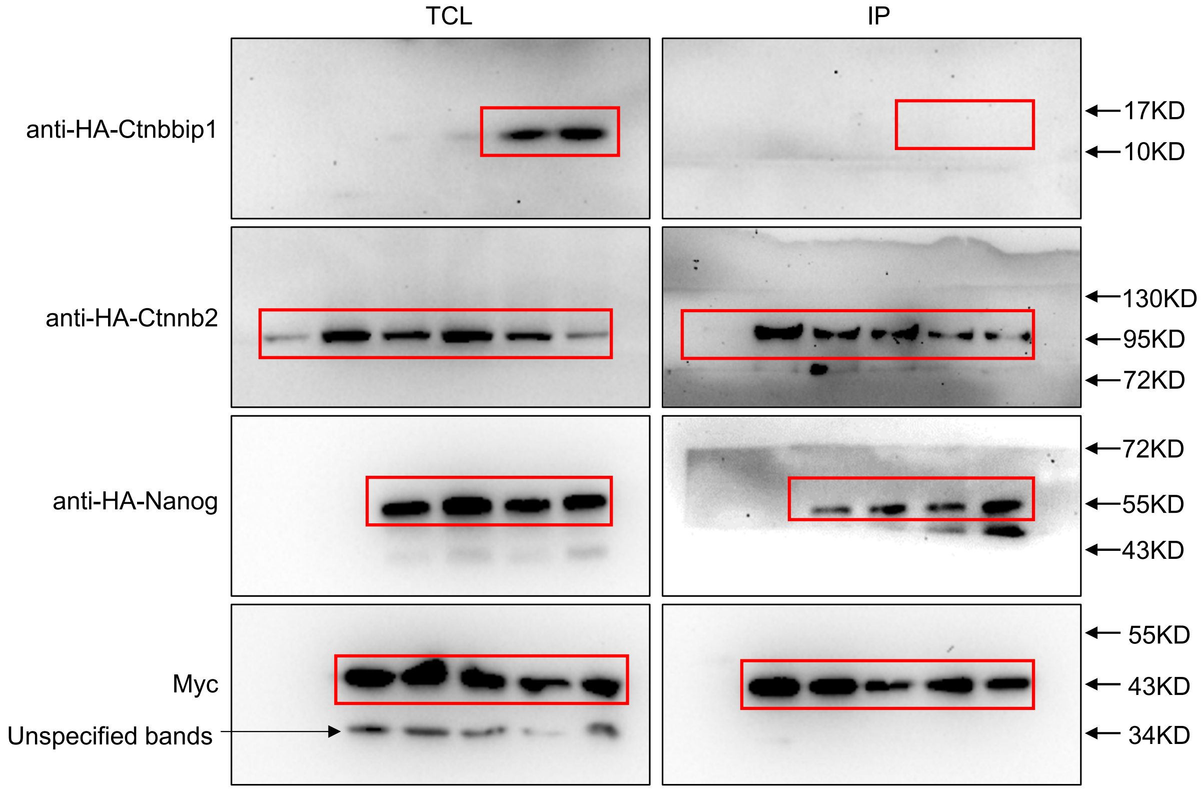

Images were acquired by ChemicDoc MP imaging system (BioRad, USA). Approximate molecular weight ladder was indicated. PVDF Membranes were cut for immunoblotting of more than one protein.

Uncropped blots for Figure 7E

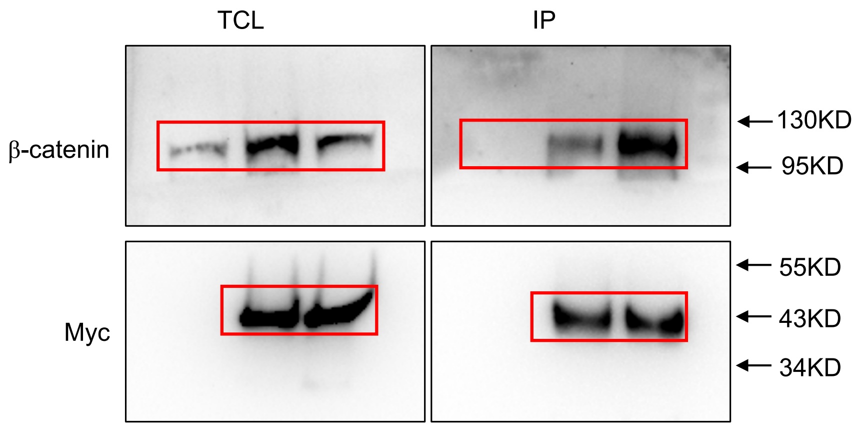

Images were acquired by ChemicDoc MP imaging system (BioRad, USA). Approximate molecular weight ladder was indicated. PVDF Membranes were cut for immunoblotting of more than one protein.

Uncropped blots for S10A Fig

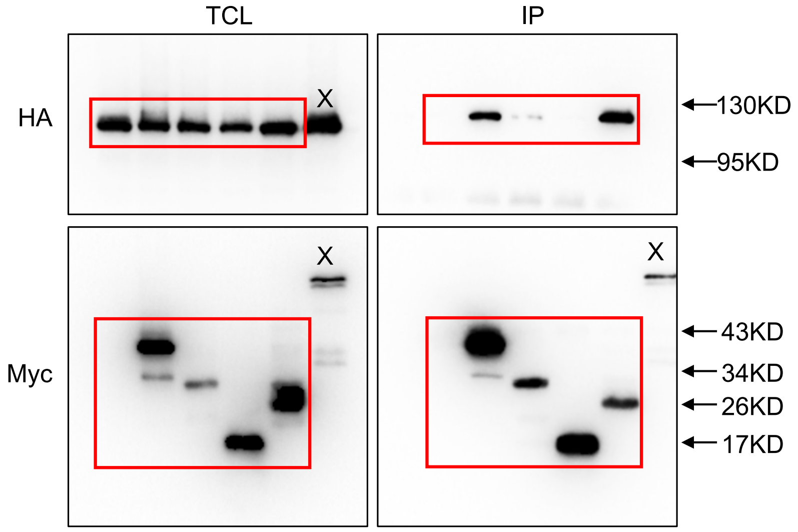

Uncropped blots for S10B Fig

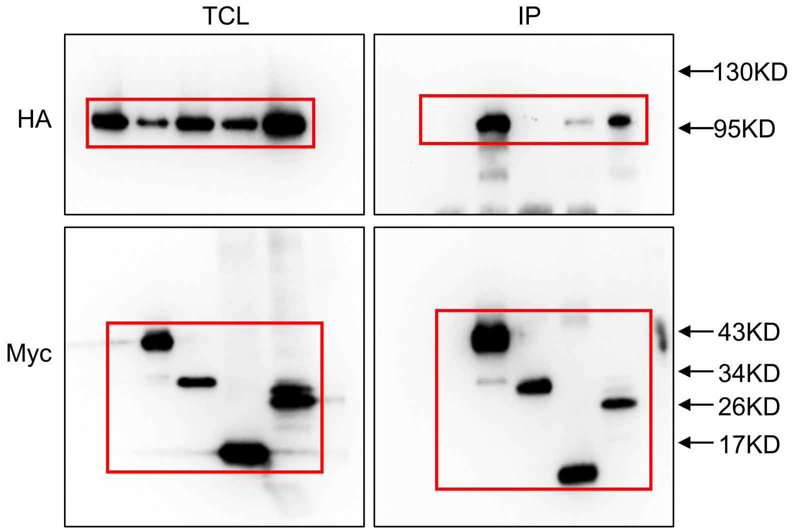

Uncropped blots for S10C Fig

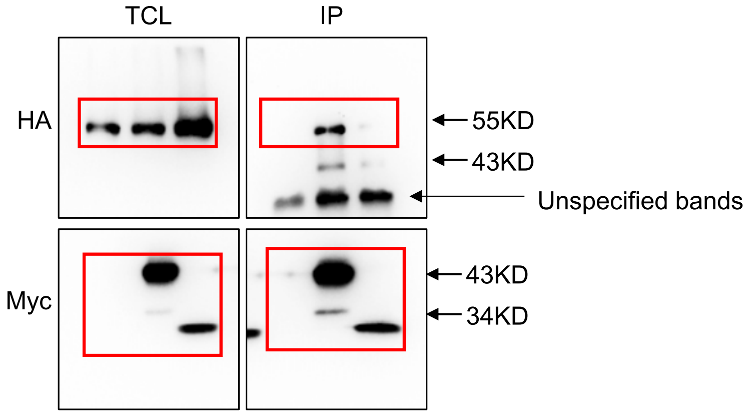

Images were acquired by ChemicDoc MP imaging system (BioRad, USA). Approximate molecular weight ladder was indicated. PVDF Membranes were cut for immunoblotting of more than one protein.
