# Supplementary material for: Nanog safeguards early embryogenesis against global activation of maternal β-catenin activity by interfering with TCF factors
Source: PLoS Biol. 2020 Jul 23;18(7):e3000561. doi: 10.1371/journal.pbio.3000561 (PMC7402524; doi:10.1371/journal.pbio.3000561)
Supplement: S1 Table — RT-qPCR, reverse-transcription quantitative PCR. (DOCX) [file pbio.3000561.s012.docx]

**S1 Table. primers used in qRT-PCR and mutant screening.**

| Primers | Sequence (5’-3’) | purpose |
| --- | --- | --- |
| tle2a-RT-F | CACCCAGGATTGGCTTTG | qRT-PCR |
| tle2a-RT-R | CTCCCTTTGTCTCCGTTTG | qRT-PCR |
| tle3a-RT-F | TGGCCTCGTCTGGCAGTA | qRT-PCR |
| tle3a-RT-R | CGAAGGGAGTTGGGTAAGTG | qRT-PCR |
| tle3b-RT-F | GGACCTTCATAACCAAACCC | qRT-PCR |
| tle3b-RT-R | TTCCCACAGCCAGCCACT | qRT-PCR |
| chd-RT-F | ATACGCCTGCTGCCATAC | qRT-PCR |
| chd-RT-R | GGCGTCTTCGCTCTTGCT | qRT-PCR |
| boz-RT-F | GATGTACTGCTGCTGCGTTCC | qRT-PCR |
| boz-RT-R | CTGCTCCGTCTGGTTGTCG | qRT-PCR |
| six3b-RT-F | TGCCAAAAACAGGCTTCAGCA | qRT-PCR |
| six3b-RT-R | CTG ACA TGG AGC GCA GAC T | qRT-PCR |
| emx1-RT-F | ACACGCTGCTACTACACGG | qRT-PCR |
| emx1-RT-R | TACTTGGTCCTGCGGTTC | qRT-PCR |
| sp5l-RT-F | ATTTCTTACAGGACCGCAC | qRT-PCR |
| sp5l-RT-R | CACGGTGAAGGTCATCTGGT | qRT-PCR |
| dkk1b-RT-F | GCTCAACTCCAACGCTATT | qRT-PCR |
| dkk1b-RT-R | ACCGCATTCCTCATCACT | qRT-PCR |
| frzb-RT-F | AACCCAGACTTCCCAATG | qRT-PCR |
| frzb-RT-R | CGAGGGATGTTGACCAGAG | qRT-PCR |
| wnt8a_ORF1-F | GCGTCGTTGGTTATGTCT | qRT-PCR |
| wnt8a_ORF1-R | AACTCCAGCAGCACTTATAG | qRT-PCR |
| wnt8a_ORF2-F | GGAGGATGTAGCGACAAC | qRT-PCR |
| wnt8a_ORF2-R | TTGCCAATCTCACGGAAG | qRT-PCR |
| mxtx2-RT-F | AAGACATGTGGACTGACTGCATTG | qRT-PCR |
| mxtx2-RT-R | GGCTCTCTTGTTCTGGAACCATAC | qRT-PCR |
| blf-RT-F | GAGTGATCCAGAAACCAGCATAATGG | qRT-PCR |
| blf-RT-R | TCACGTGAGCAGTAAGGCCAC | qRT-PCR |
| sod1-RT-F | GGTGACAACACAAACGGCTG | qRT-PCR |
| sod1-RT-R | TGGCATCAGCGGTCACATTA | qRT-PCR |
| bmp2b-RT-F | CAGCAGAGCAAACACGATAC | qRT-PCR |
| bmp2b-RT-R | TACTGGCATCTCCGAGAACT | qRT-PCR |
| bmp7-RT-F | TGCAGCTCTTAGTGGAGACC | qRT-PCR |
| bmp7-RT-R | AAACGGCTGCTTATTCTGAG | qRT-PCR |
| vent-RT-F | GGAGAGTGATGACAGTGAAGTAGA | qRT-PCR |
| vent-RT-R | ACAGCGGGATAGAGGAAGT | qRT-PCR |
| admp-RT-F | CTGTAGCCAAGAGTGAGAAG | qRT-PCR |
| admp-RT-R | CCCAGCCAGTAGAGTGAAT | qRT-PCR |
| radar-RT-F | ACTCCGCCGCTGAGAAAC | qRT-PCR |
| radar-RT-R | GAAATATCCTTAATTCAGCACC | qRT-PCR |
| nanog-WT-F1 | acccatcttatcatgcatat | mutant screening |
| nanog-TALEN-R2 | tatcgcgtcgagtgtacgcatg | mutant screening |
| tle3b-cas9-F1 | ACGGACTGCTCTGAAAGG | mutant screening |
| tle3b-cas9-R1 | TTGGCTCACATCACAAACTA | mutant screening |
| tle3a-cas9-F1 | CGGTTCTGATTGCTGTGG | mutant screening |
| tle3a-cas9-R1 | ACTTGCTGTTGGTGCTGA | mutant screening |
