# Supplementary material for: Nanog safeguards early embryogenesis against global activation of maternal β-catenin activity by interfering with TCF factors
Source: PLoS Biol. 2020 Jul 23;18(7):e3000561. doi: 10.1371/journal.pbio.3000561 (PMC7402524; doi:10.1371/journal.pbio.3000561)
Supplement: Attachment — Submitted filename: R1 P2P.doc [file pbio.3000561.s014.doc]

**Revision summary and Point-by-Point Response to Reviewers**

1. **Summary of major comments from the reviewers.**

Based on the requests and comments from the reviewers, we have performed further analyses and additional experiments. Our major revision and new supporting data are summarized and listed in the following Table 1.

**Table 1. Revision summary and new supporting data to address major comments from the reviewers.**

| **Questions** | | **Reviewers** | **Clarification on the original submission data and New data supporting** |
| --- | --- | --- | --- |
| 1 | The dynamics of beta-catenin in nuclei of non-dorsal cells should be examined. Fig. 5f shows no nuclear beta-catenin accumulation in the ventral part at 3 hpf, but its accumulation in dorsal blastomeres is apparent at 128-cell stages. How does this difference arise? | 1#: Q3 | We have carefully determined the dynamics of nuclear beta-catenin from 128-cell stage (2.25 hpf) to high stage (3.3 hpf) and presented the results in S1 Figure, also see Response new Figure 1. Our results show that the nuclear accumulation of beta-catenin starts at as early as 128-cell stage, and the dorsal nuclear accumulation starts to be prominent at 512-cell stage and the non-dorsal presence of nuclear beta-catenin is also apparent at this stage. Therefore, we present the results of beta-catenin accumulation at 512-cell stage in the main figures (see revised Fig. 1A and Fig. 5F). |
| 2 | The authors claimed that Nanog could suppress maternal beta-catenin in non-dorsal cells. Given that Nanog is maternally expressed in all blastomeres, how is its inhibitory role in dorsal blastomeres prevented? | 1#: Q4 | The maternal beta-catenin activity is strongly related to the amount of nuclear accumulation of beta-catenin. In our model (Fig. 8F), in the non-dorsal cells, the amount of nuclei-deposited maternal Nanog (red cartoon object, big size) is much higher than that of the nuclear beta-catenin (green cartoon object, small size), therefore the maternal beta-catenin activity is suppressed in the non-dorsal cells. Whereas, in the dorsal cells, due to the high amount accumulation of beta-catenin, the amount of nuclei-deposited maternal Nanog (red cartoon object) is much lower than that of the nuclear beta-catenin (green cartoon object, big size), therefore the maternal beta-catenin activity is activated in the dorsal cells. We have discussed this point in the revision (P26-27, L555-566) and clearly clarified this in the figure caption of Figure 8F. |
| 3 | mRNA rescue experiments should be performed to confirm the specific phenotypes, especially in the morphants. And Real-time PCR should be performed to further confirm ISH results in Figs. 2g, 2i, 2l, 3c, 6a, 6d and 8b. | 1#: Q5 | We have provided the mRNA rescue of MZ*nanog* in the original Fig. 6. And according your instruction, we have provided the mRNA rescue of morphants in the revised S4 Figure (see Response new Figure 2). And Real-time PCR have been performed and presented in the revised Fig. 2G, 2J, 3E, 3H, 4H, 6C and 8D. |
| 4 | In Fig.7c, they show decreased binding of β-catenin and TCF7 when Nanog is simultaneously transfected. But in the lanes 2-4 of Fig. 7d, beta-catenin/TCF7 complex was increased in the presence of Nanog compare to the case in absence of Nanog. This experiment should be repeated. In addition, the authors should check TCF7/β-catenin interaction after knockdown of nanog or in MZ*nanog* mutants to further confirm this competition mechanism. | 1#: Q6 | According to your instruction, we have repeated the experiment related to Fig. 7D and provided the revised Fig. 7D. We also conducted the TCF7/beta-catenin interaction assay in the MZ*nanog* mutants. The result showed increased amount of endogenous β-catenin was co-immunoprecipitated with Tcf7 in the absence of Nanog, which is consistent with the in vitro study. The new results were provided in revised manuscript Fig. 7E and 7F (see Response new Figure 3). |
| 5 | In the introduction, the authors completely ignore a solid part of published zebrafish research, which does not fit to their conclusions. Namely, it is firmly established that maternal and zygotic beta-catenin signaling acts in the opposing directions: zygotic Wnt represses the organizer, acting in parallel with BMP signaling. The zebrafish genome contains two β-catenin genes, β-catenin-2 and β-catenin-1. The formation of the dorsal organizing center depends on maternal expression of β-catenin-2, while both β-catenins repress the organizer and promote ventral development zygotically (Bellipanni et al. 2006). Bozozok/dharma and Chordin are repressed by zygotic Wnt through its targets, ventral transcriptional repressors Vox, Vent and Ved (Melby et al. 1997; Kawahara and Dawid 2000; Ramel and Lekven 2004; Ramel et al. 2005; Varga et al. 2007). In Maternal-Zygotic Nanog mutants, Vox, Vent as well as BMP2 are strongly downregulated, which is documented in three publications, cited by authors (Fig.7 in ref.42; Fig.3 A,B in ref. 41; Fig.4 in ref.52). In the He et al. manuscript, increase in chd and boz in Nanog mutants or morphants is iteratively referred to as a single in-vivo evidence that maternal beta-catenin signaling levels are increased upon Nanog loss-of function (lane 201, 208,209, 250, 289,301,Fig.2g,h, Fig.4 c,d,g,e, discussion). The alternative and sufficient explanation for increase in the organizer genes and for the dorsalized phenotype of the Nanog mutants is a documented decrease in zygotic b-cat activity targets and BMPs. This explanation is directly the opposite to what the authors conclude from their experiments, it should be discussed and should not be simply ignored. | 2#: Q1 | Thank you for pointing out this important issue. We have carefully discussed the control of dorsoventral patterning by maternal and zygotic beta-catenin signaling by citing the references that the reviewer has provided (P17-18, L362-382).  It has been shown that maternal and zygotic Wnt/β-catenin signals play opposing roles in regulating early dorsoventral patterning of zebrafish [1]. The dorsal organizing center formation depends on the maternal expression of *ctnnb2*, whereas both *ctnnb1* (*β-catenin1*) and *ctnnb2* are required for repressing the dorsal organizer genes, *boz* and *chd*, via the zygotic Wnt/β-catenin targets, *vox*, *vent* and *ved* [2-6]. Therefore, we were curious whether the dorsalization of MZ*nanog* embryos was due to activation of maternal β-catenin signaling. We tried to rescue the MZnanog embryos through interfering the expression of *ctnnb1* or *ctnnb2* by MO-mediated knockdown. Morphologically, the developmental defects of MZ*nanog* at gastrula stage was largely rescued by knockdown of *ctnnb*2, but not *ctnnb1* (revised MS Fig 4E, also see Response new Figure 4). At molecular level, knockdown of *ctnnb2* reduced the ectopic expression of *boz* and *chd*, but ctnnb1 knockdown did not (revised MS Fig 4F and 4G, also see Response new Figure 4), which was further confirmed by RT-qPCR analysis (revised MS Fig 4H, also see Response new Figure 4). As a result, the expression reduction of ventrally expressed *bmp2b*, *bmp7* and *vent* could be restored by knockdown of maternally expressed *ctnnb2* but not zygotically expressed *ctnnb1* (revised MS S6A Fig, also see Response new Figure 4). TOPflash assay also showed that the elevation of β-catenin signaling activity in MZ*nanog* embryos at 4 hpf could be significantly reduced by knockdown of *ctnnb2* but not *ctnnb1* (revised MS Fig 4I, also see Response new Figure 4). Therefore, we conclude that loss of maternally provided Nanog activity leads to hyperactivation of maternal β-catenin activity and dorsalization, which is mediated by *ctnnb2*. |
| 6 | Alternatively, the forebrain defects may be an effect of VP-16 fusion. Two additional experimental controls are absolutely necessary to distinguish between the possibilities: rescuing MZ*nanog* with 1) full-length Nanog – VP16 fusion and 2) N-terminally truncated Nanog. If N-terminus of Nanog is required for embryo development, full-length-VP16 would completely rescue the MZ*nanog* embryos, and N-terminal truncation will produce the embryos with forebrain defects (or it would not rescue at all). If otherwise, the in-vitro interaction between TCF7 and Nanog is dispensable for development. | 2#: Q3 | We have performed complemental experiments by overexpression of full-length nanog-VP16 and N-terminal truncated Nanog in MZ*nanog*. In consistent to what was proposed by the reviewer, the full-length nanog-VP16 showed the same rescue effect as the full-length nanog, and N-terminally truncated Nanog showed no rescue effect (revised manuscript S8 Fig., also see Response new Figure 5). In addition, we performed in vivo interaction assay of TCF7 and beta-catenin in WT and MZ*nanog*, the result showed increased amount of endogenous β-catenin was co-immunoprecipitated with Tcf7 in the absence of Nanog, which is consistent with the in vitro study. The new results were provided in revised manuscript Fig. 7E and 7F (also see Response new Figure 3). |
| 7 | At least one Groucho homolog, tle2a is expressed maternally in a high level, even exceeding tle3a and tle3b (refs: Thisse 2001, zfin.org, and “A high-resolution mRNA expression time course of embryonic development in zebrafish”, White et al., Elife, 2017). To substantiate and validate their statement, the authors should knock down at three Grouchos (i.e. by injecting tle2a Moropholino to the double MZtle3a/b mutant); and show that the other members (tle2b, tle2c and tle5) are not expressed maternally. Otherwise, the not conclusive statement can be removed. | 2#: Q4 | We have compared the function domain of all zebrafish Tle genes (revised manuscript S3A Fig, also see Response new Figure 6), found that *tle2a*, *tle3a* and *tle3b* have the possibility to repress the maternal β-catenin activity. We further checked the maternal expression of *tle2a*, *tle3a* and *tle3b* (revised manuscript Fig 1C and 1D, also see Response new Figure 6) and knockdown of all this three Tles did not interrupt the early embryonic development (revised manuscript S3D-3F Fig, also see Response new Figure 6). We agree with the reviewer that our study could not exclude the possibility that a combination of all TLEs contributes the repression of maternal β-catenin activity in zebrafish. Therefore, we tuned down our conclusion by stating that “Maternally expressed TLEs do not likely repress maternal β-catenin activity in non-dorsal cells of zebrafish early embryo” (P8 L165-166). |
|  | Perez-Camps et al (ref.52) who injected the very same morpholino in slightly different concentrations (1.6 ng/embryo and 0.4 ng/embryo), saw the forebrain defects only in the higher dose, in parallel with other multiple defects. This issue has to be discussed. | 2#: Q6 | For *nanog* knockdown experiment, we repeated many times and the results are always consistent. Injection of *nanog* MO at moderate concentration (1.6 ng/embryo) resulted in hyper-dorsalized phenotype, which is identical to the result of Perez-Camps et al [7], a publication from the Reviewer 3’s lab. Injection of *nanog* MO at low dose (0.4 ng/embryo) resulted in forebrain defect, which could be rescued by knockdown of *wnt8a*. Moreover, in the revised MS, we proved the specificity of these two types of phenotypes resulting from MO injection, which can be rescued by overexpression of MO-mismatched nanog mRNA (S4 Figure in revised manuscript, also see Response new Figure 2). |
|  | Based on the authors conclusion, what would be the effect of removing endogenous maternal beta-Catenin via MO depletion in MZ null null Nanog. To what extent will this rescue the phenotype. This is a more physiological assay than overexpression of gsk3b or ck1a. | 3#: Q1 | We knocked down the maternally expressed *beta-catenin* 2 in MZ*nanog* embryos (presented in revised manuscript Fig 4E-4I, also see Response new Figure 4), and showed the rescue of hyper-dorsalization in MZ*nanog* (revised manuscript Fig. 4E and 4F). |
|  | The endogenous levels of expressions of Bmp2/7 and Admp by QPCR and WISH should be documented. In fact, one ligand that would be key to assess is the maternal TGFbeta Radar. Such data should be included in the revised manuscript to better describe the observed DV defects. | 3#: Q3 | We detected the expression level of *bmp2b*, *bmp7* (S6A Fig in revised manuscript, also see Response new Figure 4), *admp* and *radar* (S6B Fig in revised manuscript, also see Response new Figure 7) in MZ*nanog* by RT-qPCR. The BMP signaling activity was reduced in MZ*nanog*, and this reduction can be rescued by knockdown of *ctnnb2*, further confirmed the dorsalized phenotype of MZ*nanog* is owing to the hyperactivation of maternal beta-catenin activity. Admp is a BMP receptor that suppresses dorsal and anterior structures and its expression is maintained by [Nodal signaling](https://www.sciencedirect.com/topics/biochemistry-genetics-and-molecular-biology/nodal-signaling)[8]. Due to the down-regulation of Nodal activity [9], the expression of *admp* is significantly reduced in MZ*nanog*. The increased expression of *radar* in MZ*nanog* at 2 hpf might be due to a negative feedback by the ectopic activation of dorsal genes. Nevertheless, the upregulated maternal *radar* in MZ*nanog* embryos and the downregulated *admp* in MZ*nanog* are fairly different from what has been described in the classical BMP-inhibited embryos [10], indicating that the dorsalization phenotype is not caused by downregulation of BMP signaling. |

**2. Point-by-point responses to reviewers**

**Reviewer #1:**

Previous studies in zebrafish have established that maternal Nanog plays a role in clearing maternal mRNAs for midblastula transition and is also required for extraembryonic tissue development and cell variability. This manuscript investigated maternal nanog functions using maternal/zygotic nanog mutants and through biochemical experiments. It first observed that both Mnanog and MZnanog mutants exhibited dorsalized phenotypes, and then established that the dorsalized phenotype was associated with over-activation of beta-catenin signaling before midblastula transition. The authors finally uncovered the novel mechanism by which maternal Nanog binds to beta-catenin to prevent the latter from binding to the transcriptional activator TCF7/4. This study is comprehensive and the major conclusions are well supported by experimental data. The manuscript is well organized. To improve the manuscript, the following issues needed to be considered.

-Thank you very much for your appreciation of our work.

Major concerns:
1)It is now well known that cytoplasmic beta-catenin can be stabilized and translocated into the nucleus in various ways independent of Wnt ligands/Wnt receptors-mediated signaling. Thus, beta-catenin signaling is not necessarily identical to Wnt signaling. In Xenopus and zebrafish, increasing evidence suggest that Wnt ligands/Wnt receptors signaling is not activated before midblastula transition. The authors should be cautious about this issue. For example, the authors in several context mentioned hyperactivation/overactivation of maternal Wnt activity in MZnanog, and this is inappropriate.

**Response 1**: We totally agree with you on this point. We have changed all the phrase of “maternal Wnt activity” to “maternal β-catenin activity” (highlighted).

2)Fig 4A showed that Nanog protein level at the shield stage was sharply dropped compared to that at the sphere stage, but Fig. 2C showed Nanog protein level at 6 hpf (shield stage) was much higher than that at 4 hpf. This inconsistency should be confirmed or explained.

**Response 2**: Thank you for pointing out this issue. We have repeated the relevant experiments and double checked the results related to original Fig 2C and Fig 4A. The embryos stage should be 2, 3, 4 hpf in Fig 2C, and we have corrected the mistake in the revised Fig 2C.

3)The dynamics of beta-catenin in nuclei of non-dorsal cells should be examined. Fig. 5f shows no nuclear beta-catenin accumulation in the ventral part at 3 hpf, but its accumulation in dorsal blastomeres is apparent at 128-cell stages. How does this difference arise?

**Response 3**: Thank you for the comment. We have carefully determined the dynamics of nuclear beta-catenin from 128-cell stage (2.25 hpf) to high stage (3.3 hpf) and presented all the results in S1 Figure (also see Response new Figure 1). Our results show that the nuclear accumulation of beta-catenin starts at as early as 128-cell stage, and the dorsal nuclear accumulation starts to be prominent at 512-cell stage and the non-dorsal presence of nuclear beta-catenin is also apparent at this stage. Therefore, we present the results of beta-catenin accumulation for 512-cell stage in the main figures (Fig. 1A and Fig. 5F) in the revised manuscript.

Response new Figure 1. Nuclear β-catenin localizes in both dorsal and non-dorsal cells. Nuclear β-catenin distribution in zebrafish blastodermal cells was detected by whole-mount immunofluorescence from 128-cell stage to high stage. Embryos was mounted and signal was detected at animal view. Nuclei were co-stained with DAPI. At least 15 embryos were detected in each stage. Scale bar, 50 µm.

4)The authors claimed that Nanog could suppress maternal beta-catenin in non-dorsal cells. Given that Nanog is maternally expressed in all blastomeres, how is its inhibitory role in dorsal blastomeres prevented?

**Response 4**: Thank you for this critical question. The maternal beta-catenin activity is strongly related to the amount of nuclear accumulation of beta-catenin. In our model (Fig 8F), in the non-dorsal cells, the amount of nuclei-deposited maternal Nanog (red cartoon object, big size) is much higher than that of the nuclear beta-catenin (green cartoon object, small size), therefore the maternal beta-catenin activity is suppressed in the non-dorsal cells. Whereas, in the dorsal cells, due to the high amount accumulation of beta-catenin, the amount of nuclei-deposited maternal Nanog (red cartoon object) is much lower than that of the nuclear beta-catenin (green cartoon object, big size), therefore the maternal beta-catenin activity is activated in the dorsal cells. We have discussed this point in the revision (P26-27, L555-566) and clearly clarified this in the figure caption of Figure 8F.

5)The authors observed dorsalization, posteriorization and MZT defects in nanog morphants and MZnanog, mRNA rescue experiments should be performed to confirm the specific phenotypes, especially in the morphants. And Real-time PCR should be performed to further confirm ISH results in Figs. 2g, 2i, 2l, 3c, 6a, 6d and 8b.

**Response 5:** Thank you for pointing out. We have provided the mRNA rescue of MZ*nanog* in the original Fig. 6. And according your instruction, we have provided the mRNA rescue of morphants in the revised S4 Figure (see Response new Figure 2). And Real-time PCR have been performed and presented in revised Fig. 2G, 2J, 3E, 3H, 4H, 6C and 8D.

Response new Figure 2 (A) Three classes of phenotypes -- WT-like, posterization, and dorsalization, were characterized in *nanog* morphants at 36 hpf. Scale bar, 100 µm. (B) Overexpression of *nanog* mismatched mRNA (*nanog* MO targeted site is mutated) rescued the posterization, and dorsalization defect of nanog morphant, proving the specificity of *nanog* MO. N represents analyzed embryo number. The underlying data in this figure can be found in [S1 Data](https://journals.plos.org/plosbiology/article?id=10.1371/journal.pbio.3000696#pbio.3000696.s019). hpf, hours post fertilization; MO, morpholino; WT, wild type.

6)The authors propose a competition mechanism between Nanog and beta-catenin for their binding with TCF7. In Fig.7c, they show decreased binding of β-catenin and TCF7 when Nanog is simultaneously transfected. But in the lanes 2-4 of Fig. 7d, beta-catenin/TCF7 complex was increased in the presence of Nanog compare to the case in absence of Nanog. This experiment should be repeated. In addition, the authors should check TCF7/β-catenin interaction after knockdown of nanog or in MZnanog mutants to further confirm this competition mechanism.

**Response 6:** Thank you for your suggestion. We have repeated the experiment related to Fig 7D and provided the revised Fig 7D. We also conducted the TCF7/beta-catenin interaction assay in the MZ*nanog* mutants, the result showed increased amount of endogenous β-catenin was co-immunoprecipitated with Tcf7 in the absence of Nanog, which is consistent to the in vitro study. The new results were provided in revised manuscript Fig. 7E and 7F (see Response new Figure 3).

Response new Figure 3 (A) Co-immunoprecipitation assay showed that increased amount of endogenous β-catenin could interact with Tcf7 in MZ*nanog* compared with WT embryos. Embryos were collected at 4 hpf. (B) The intensity ratio of β-catenin to Tcf7 in (A). Error bars, mean ± SD, *P < 0.05. The P values in this figure were calculated by Student t test. The underlying data in this figure can be found in [S1 Data](https://journals.plos.org/plosbiology/article?id=10.1371/journal.pbio.3000696#pbio.3000696.s019). MZ*nanog*, maternal-zygotic mutant of nanog; hpf, hours post fertilization; WT, wild type.

7) Results in Fig. 5d and Fig. 5e are not consistent. The lane 2 in Fig. 5d showed obvious reduction of active beta-catenin level, while the statistic result in Fig. 5e showed no significant change. This experiment needed biological repeats.

**Response 7:** Thank you for pointing out this issue. We have repeated this experiment and recalculated the ratio of active beta-catenin vs total beta-catenin, the new result is presented in revised manuscript Fig. 5D and 5E.

Minor concerns:

1)Scale bars should be added in embryos pictures.

**Response 8:** We have added the scale bars in embryos pictures.

2)Legends for Fig. 3g is missing.

**Response 9:** We are Sorry for the mistake. We have corrected and rearranged the data.

3)Protein markers should be labeled for WB results in Fig. 2c, Fig. 4a, Fig. 7 and Fig. S5.

**Response 10:** Thank you for pointing out. We have added protein markers in revised Fig 2C, Fig 4A, Fig 5D, Fig 7 and S10 Fig (original S5 Fig).

4)Pictures in Fig. 2g (embryo stage for boz in MO group is not consistent); Fig. 4c and Fig. 6a should be improved.

**Response 11:** We have repeated experiment related to Fig 2G and changed the Fig 2G (revised Fig. 2H) with new pictures. We also improved the signal contrast of Fig 4C and Fig 6A.

5)ENH mRNA in Line 341 should be substituted by EN-Nanog.

**Response 12:** Sorry for the mistake. We have corrected it in P20 L422.

6)Grammatical mistakes in Line 46/88/257/518 should be corrected.

**Response 13:** We have corrected the grammatical mistakes.

7)Double check typos. For example, line 270, “maternal deposit” should be “maternally deposited”; line 278, “was completed” should be “was completely”; line 462, “serve ventralization” should be “severe ventralization”.

**Response 14:** Sorry for the mistake. We have corrected the mistakes.

**Reviewer #2:**

The manuscript by He et al investigates the interactions between beta-catenin signaling and homeodomain protein Nanog in zebrafish.  The novel results in this paper are mostly coming from in-vitro and overexpression assays; in-vivo Nanog loss-of function data are largely redundant with the previous publications (cited in the paper).  Using beta-catenin reporter plasmid (Topflash –luciferase reporter assay), the authors show that the beta-catenin signaling levels are elevated in zebrafish embryos upon Nanog loss-of function. Using overexpression assays in zebrafish embryos and 293 HEK cells, the authors further show that Nanog antagonizes beta-catenin-dependent activation of the reporter plasmid. They further demonstrate by co-immunoprecipitation, that N-terminal domain of Nanog is responsible for in-vitro binding to Tcf7, and may block beta-catenin-TCF7 interaction. However, from my point of view, the authors did not provide enough evidence that this in-vitro mechanism is somewhat important for vertebrate development. The global conclusions of the manuscript, claimed already in the title “Nanog safeguards early embryogenesis against global activation of maternal ß-catenin activity by interfering with TCF factors” are not at all validated by the presented results (see the major points below).  The manuscript, in its present form, cannot be published without additional extensive experimental work, which may challenge the data interpretation. Alternatively, the novel in-vitro part can be published separately in a more specialized journal.
- Thank you very much for your critical advices. We have performed additional experiments to show the importance of this interaction for in vivo development, for example, visualizing nuclear beta-catenin from 128-cell stage to high stage, knockdown of all the maternally expressed repressive TLEs, rescuing the *nanog* morphants by mismatched mRNA injection, rescuing MZ*nanog* by beta-catenin 2 MO injection, analysis of BMP ligands in MZ*nanog* the rescued embryos, rescuing MZ*nanog* by N-terminal deleted Nanog or full-length Nanog-VP16 fusion, coIP analysis in MZ*nanog* embryos, and conducting series of RT-qPCR analysis as well. According to your comments, we also thoroughly revised the manuscript by adding more experimental details and more discussions.

Major comment 1:
In the introduction, the authors completely ignore a solid part of published zebrafish research, which does not fit to their conclusions. Namely, it is firmly established that maternal and zygotic beta-catenin signaling acts in the opposing directions:  zygotic Wnt represses the organizer, acting in parallel with BMP signaling. The zebrafish genome contains two β-catenin genes, β-catenin-2 and β-catenin-1. The formation of the dorsal organizing center depends on maternal expression of β-catenin-2, while both β-catenins repress the organizer and promote ventral development zygotically (Bellipanni et al. 2006). Bozozok/dharma and Chordin are repressed by zygotic Wnt through its targets, ventral transcriptional repressors Vox, Vent and Ved (Melby et al. 1997; Kawahara and Dawid 2000; Ramel and Lekven 2004; Ramel et al. 2005; Varga et al. 2007). In Maternal-Zygotic Nanog mutants, Vox, Vent as well as BMP2 are strongly downregulated, which is documented in three publications, cited by authors (Fig.7 in ref.42; Fig.3 A,B in ref. 41;  Fig.4 in ref.52). In the He et al. manuscript, increase in chd and boz in Nanog mutants or morphants is iteratively referred to as a single in-vivo evidence that maternal beta-catenin signaling levels are increased upon Nanog loss-of function (lane 201, 208,209, 250, 289,301,Fig.2g,h, Fig.4 c,d,g,e, discussion).  The alternative and sufficient explanation for increase in the organizer genes and for the dorsalized phenotype of the Nanog mutants is a documented decrease in zygotic b-cat activity targets and BMPs. This explanation is directly the opposite to what the authors conclude from their experiments, it should be discussed and should not be simply ignored.

**Response 15**: Thank you for pointing out this important issue. We have carefully discussed the control of dorsoventral patterning by maternal and zygotic beta-catenin signaling by citing the references that the reviewer has provided (P17-18, L362-382).

It has been shown that maternal and zygotic Wnt/β-catenin signals play opposing roles in regulating early dorsoventral patterning of zebrafish [1]. The dorsal organizing center formation depends on the maternal expression of *ctnnb2* (*β-catenin2*), whereas both *ctnnb1* (*β-catenin1*) and *ctnnb2* are required for repressing the dorsal organizer genes, *boz* and *chd*, via the zygotic Wnt/β-catenin targets, *vox*, *vent* and *ved* [2-6]. Therefore, we were curious whether the dorsalization of MZ*nanog* embryos was due to activation of maternal β-catenin signaling. We tried to rescue the MZnanog embryos through interfering the expression of *ctnnb1* or *ctnnb2* by MO-mediated knockdown. Morphologically, the developmental defects of MZ*nanog* at gastrula stage was largely rescued by knockdown of *ctnnb*2, but not *ctnnb1* (revised MS Fig 4E, also see Response new Figure 4). At molecular level, knockdown of *ctnnb2* reduced the ectopic expression of *boz* and *chd*, but *ctnnb1* knockdown did not (revised MS Fig 4F and 4G, also see Response new Figure 4), which was further confirmed by RT-qPCR analysis (revised MS Fig 4H, also see Response new Figure 4). As a result, the expression reduction of ventrally expressed *bmp2b*, *bmp7* and *vent* could be restored by knockdown of maternally expressed *ctnnb2* but not zygotically expressed *ctnnb1* (revised MS S6A Fig, also see Response new Figure 4). TOPflash assay also showed that the elevation of β-catenin signaling activity in MZ*nanog* embryos at 4 hpf could be significantly reduced by knockdown of *ctnnb2* but not *ctnnb1* (revised MS Fig 4I), also see Response new Figure 4). Therefore, we conclude that loss of maternally provided Nanog activity leads to hyperactivation of maternal β-catenin activity and dorsalization, which is mediated by *ctnnb2*.

Response new Figure 4 (A) Knockdown ctnnb2 but not ctnnb1 rescued the developmental defects of MZnanog at gastrula stage. The numbers below the morphology pictures mean number of embryos showing representative phenotype/total number of embryos. Scale bar, 500 µm. (B) WISH analysis showing expression of chd and boz were expanded in MZnanog embryos, and knockdown of ctnnb2 (β2 MO) but not ctnnb1 (β1 MO) rescued these defects. boz was detected at 4 hpf, chd was detected at 5 hpf. Scale bar, 100 µm. (C) Statistical analysis of the embryos in panel (B). N represents analyzed embryo number. (D) Relative mRNA level of boz and chd in the embryos of WT, MZnanog, MZnanog co-injected with ctnnb1 MO (+β1 MO) and MZnanog co-injected with ctnnb2 MO (+β2 MO). Error bars, mean ± SD, **P < 0.01, NS means no significant difference. (E) TOPflash assay showing β-catenin transcriptional activity was significantly up-regulated in the embryos of MZnanog compared with WT, and co-injection of ctnnb2 MO (+β2 MO) but not ctnnb1 MO (+β1 MO) into MZnanog embryos significantly decreased the transcriptional activity. Error bars, mean ± SD, **P < 0.01, ***P < 0.001. (F) Relative mRNA level of bmp2b, bmp7 and BMP target vent, were reduced in MZnanog examined by RT-qPCR analysis Depletion of ctnnb2 could restore the abnormal expression of bmp2b, bmp7, and vent, whereas knockdown of ctnnb1 could not. bmp2b, bmp7 and vent was detected at 6 hpf. Error bars, mean ± SD, **P < 0.01, NS means no significant difference. The P values in this figure were calculated by Student t test. The underlying data in this figure can be found in [S1 Data](https://journals.plos.org/plosbiology/article?id=10.1371/journal.pbio.3000696#pbio.3000696.s019). MZnanog, maternal-zygotic mutant of nanog; MO, morpholino; WISH, whole-mount in situ hybridization; RT-qPCR, reverse-transcription quantitative PCR; hpf, hours post fertilization; WT, wild type.

Major comment 2:

Related to comment 1: In-vitro, the authors measured endogenous beta-catenin activity using beta-catenin-responsive Topflash plasmid (TCF1 binding sites fused to the luciferase reporter gene).  The authors show that beta-catenin- induced activity of luciferase in zebrafish embryos (Fig.3e, f,g; Fig.4f). It is absolutely unclear to me, why the authors interpret their results as an increase of maternal beta-catenin activity (lane 250 on): DNA, injected into the early embryo, can be only transcribed after ZGA, the luciferase is measured at 8 hpf, late gastrula stage. At best, the authors measure some complex mixture of maternal and zygotic activities of both beta-catenins (provided that luciferase half-life is 3-4 hours). The fact, that there is more unbound nuclear beta-catenin in MZnanog, can be interpreted in two different ways. The authors suggest, that Nanog represses the binding of maternal beta-catenin to TCFs by means of N-terminus, blocking DNA-bound TCFs. Alternative interpretation, for the embryos, compatible with zygotic repression of Vent Vox and Ved genes in MZnanog (refs 41,42,52)- would be that in the ventral cells of MZnanog, zygotic beta-catenin cannot efficiently access its targets on DNA without Nanog pioneering activity, so that excess of beta-cat remains in the nucleus and activates the ectopic plasmid. In fact, considering the results in 293 HEK cells, both explanations can be true. This issue has to be discussed.

**Response 16**: Thank you for pointing out this important issue. In the revised manuscript, we carefully examined and challenged conclusion that endogenous maternal beta-catenin activity is increased in *nanog* morphants and MZ*nanog* embryos. First, we performed TOPflash assay at 4 hpf in *nanog* morphants and MZ*nanog*, the results showed significant increase of TOPflash activity in *nanog* morphants or MZ mutant at 4 hpf (Fig.3I, 4I and 8E). Second, we used *beta-catenin 2* MO to remove the maternal beta-catenin activity to rescue MZ*nanog* embryos (Fig 4E-4I). Third, TOPflash assay in 293T cells clearly demonstrated that the downregulated beta-catenin transcriptional activity was due to increased expression of Nanog (Fig 3 J, K). Fourth, we conducted coIP analysis in MZ*nanog* embryos and showed the interaction between Nanog/Tcf7/beta-catenin in an in vivo condition (Fig 7E, F).

Major comment 3:
Figure 6C, lane 330 – on. The authors claim that N-terminus of Nanog is required for supression of beta-catenin activity, based on the MZnanog rescue experiments. They show, that all gastrulation defects in MZnanog can be completely rescued by the full-length protein, c-terminal-truncated Nanog, and by N-terminal truncated Nanog, fused to VP-16 transcriptional activating domain (Nanog-VP16).  However, the first two constructs rescue the embryos to full viability, while the injection of N-terminal truncated Nanog, fused to VP-16 results in posteriorized embryos with forebrain defects.  The authors claim that forebrain defects result from the absence of Nanog N-terminus in Nanog-VP16. Alternatively, the forebrain defects may be an effect of VP-16 fusion. Two additional experimental controls are absolutely necessary to distinguish between the possibilities: rescuing MZnanog with 1) full-length Nanog – VP16 fusion and 2) N-terminally truncated Nanog. If N-terminus of Nanog is required for embryo development, full-length-VP16 would completely rescue the MZnanog embryos, and N-terminal truncation will produce the embryos with forebrain defects (or it would not rescue at all). If otherwise, the in-vitro interaction between TCF7 and Nanog is dispensable for development.

**Response 17**: According to your suggestion, we performed complemental experiments by overexpression of full-length *nanog*-VP16 and N-terminal truncated Nanog in MZ*nanog*. In consistent to what was proposed by the reviewer, the full-length *nanog*-VP16 showed the same rescue effect as the full-length *nanog*, and N-terminal truncated Nanog showed no rescue effect (revised manuscript S8 Fig, also see Response new Figure 5). In addition, we performed in vivo interaction assay of TCF7 and beta-catenin in WT and MZ*nanog*, the result showed that increased amount of endogenous β-catenin was co-immunoprecipitated with Tcf7 in the absence of Nanog, which is consistent with the in vitro study. The new results were provided in revised manuscript Fig. 7E and 7F (also see Response new Figure 3).

Response new Figure 5 Overexpression of *nanog*_FL and vp16-*nanog*_FL could fully rescue the developmental defect of MZnanog, and N-terminal truncated nanog (*nanog*_ΔN) could not. Scale bar, 100 µm. nanog_FL, full length of Nanog; *nanog*_ΔN, N-terminal truncated Nanog; vp16-*nanog*_FL, full length of Nanog fusion with Vp16.

Major comment 4:
(first chapter of the results, line 133 on). Groucho is known transcriptional repressor of beta-catenin activity, first found in Drosophila. Vertebrate genomes contain multiple homologs of Groucho: for instance, Zebrafish genome has 6 homologs: tle2a, tle2b, tle2c, tle3a, tle3b and tle5 (zfin.org).   The authors knocked down two out of 6 zebrafish Grouchos, tle3a and tle3b. They do not see the developmental defects in single and double mutants, which allows them to conclude that: “Tle3a and Tle3b do not contribute to the repression of maternal beta-catenin activity”.  To my opinion, the conclusion is misleading: is well possible that Tle3a and Tle3b contribute redundantly to the repression of the maternal b-cat 2 activity together with the other maternal Grouchos. At least one Groucho homolog, tle2a is expressed maternally in a high level, even exceeding tle3a and tle3b (refs: Thisse 2001, zfin.org, and “A high-resolution mRNA expression time course of embryonic development in zebrafish”, White et al., Elife, 2017). To substantiate and validate their statement, the authors should knock down at three Grouchos (i.e. by injecting tle2a Moropholino to the double MZtle3a/b mutant); and show that the other members (tle2b, tle2c and tle5) are not expressed maternally. Otherwise, the not conclusive statement can be removed.

**Response 18:** Thank you for your instruction. We have compared the function domain of all zebrafish Tle genes, and found that *tle2a*, *tle3a* and *tle3b* encode long Groucho/TLEs which have the possibility to repress the maternal β-catenin activity (revised manuscript S3A Fig, P10 L192-204, also see response new Figure 6). We further checked and compared the maternal expression of *tle2a*, *tle3a* and *tle3b* (revised manuscript Fig. 1C and 1D, P10 L207-210, also see response new Figure 6) and knockdown of all this three Tles did not interrupt the early embryonic development (revised manuscript S3D-3F Fig, P11 L215-218, also see Response new Figure 6). We agree with the reviewer that our study could not exclude the possibility that a combination of all TLEs contributes the repression of maternal β-catenin activity in zebrafish. Therefore, we tuned down our conclusion by stating that “Maternally expressed TLEs do not likely repress maternal β-catenin activity in non-dorsal cells of zebrafish early embryo” (P8 L165-166).

Response new Figure 6 (A) Functional domain analysis of zebrafish Tle2a, Tle2b, Tle2c, Tle3a, Tle3b and Tle5. (B) In situ hybridization on cryosections of ovaries and WISH analysis of unfertilized eggs (pink framed squares) showing *tle2a*, *tle3a* and *tle3b* are maternally expressed during oogenesis and in unfertilized eggs. Scale bar, 100 µm. (C) In comparison with *tle2a*, *tle3a* and *tle3b* are significantly highly expressed in matured eggs as shown by RT-qPCR analysis. Error bars, mean ± SD, *P < 0.05, **P < 0.01. (D) Validation of *tle2a* MO effectiveness. *tle2a* MO target sequence was fused with GFP to result in pTle2a-GFP construct. pTle2a-GFP construct or pTle2a-GFP combination with *tle2a* MO was injected at one-cell stage. Fluorescence was observed at 10 hpf. Scale bar, 500 µm. (E) Knockdown of *tle2a* at 2 ng/embryo did not affect the early development of zebrafish. Scale bar, 500 µm. (F) Knockdown of *tle2a* (2 ng/embryo), *tle3a* (2 ng/embryo) and *tle3b* (2 ng/embryo), respectively or combination, does not lead to early embryonic development defect at 10 hpf and 56 hpf. MO or combined MOs was injected at one-cell stage and at least 50 embryos were injected and observed. Scale bar, 500 µm. hpf, hours post fertilization; MO, morpholino.

Minor comments:

Figure legends for Fig.4h and Fig.8a: (lanes 1023-1025 and 1093-1096). Figure legend 4h (lanes 1023-1025) states the  rescue of “the early developmental defect of MZnanog”  Figure legend 8a (lanes 1093-1096) states the rescue of : “the dorsalization phenotype of MZnanog”.    Menawhile, both figures show similar phenotypes, which only with with a lot of good will can be interpreted as a partial epiboly rescue in MZnanog by indicated constructs. Very confusing!
**Response 19:** Sorry for the ambiguity. In the revised MS, at morphological level, we concluded that the early developmental defect at gastrula stage of MZ*nanog* was rescued (P18 L371, P26 L545, P61 L1421, P66 L1534-1535). And from the view of dorsal marker expression, we defined that the hyper-dorsalization phenotype of MZ*nanog* was rescued. We have corrected the description.

Lane 172 on – “knockdown of nanog leads to dorsalization and posteriorisation”. Here, the authors inject the previously published Nanog morpholino, used in two studies (39 and 52). They find, that the moderate dose of the morpholino (1.4 ng/embryo) results in dorzalisation of the embryos, while the head is normal:  the low dose (0.5 ng/embryo) results in forebrain defects, which are similar to wnt8 overexpression.  Strange enough, Perez-Camps et al (ref.52) who injected the very same morpholino in slightly different concentrations (1.6 ng/embryo and 0.4 ng/embryo), saw the forebrain defects only in the higher dose, in parallel with other multiple defects. This issue has to be discussed.

**Response 20:** Thank you for the questions. For *nanog* knockdown experiment, we repeated many times and the results are always consistent. Injection of *nanog* MO at moderate concentration (1.6 ng/embryo) resulted in hyper-dorsalized phenotype, which is identical to the result of Perez-Camps et al [7], a publication from the Reviewer 3’s lab. Injection of *nanog* MO at low dose (0.4 ng/embryo) resulted in forebrain defect, which could be rescued by knockdown of *wnt8a*. Moreover, in the revised MS, we proved the specificity of these two types of phenotypes resulting from MO injection, which can be rescued by overexpression of MO-mismatched nanog mRNA (S4 Figure in revised manuscript, also see Response new Figure 2).

In Materials and Methods (595-602), the tcf4 mRNA used in the manuscript is missing, mxtx2, which was not used, is present.

**Response 21:** Sorry for the mistakes. We have added the *tcf4* (*tcf7l2*) mRNA (P35 L724) and deleted the *mxtx2* mRNA injection in Materials and Methods.

Lane 275 on: References to zebrafish Tcf7 and Tcf4 is not provided; it is unclear which genes were actually used. Zebrafish tcf4 is first expressed in organogenesis, tcf7 is expressed maternally but does not have known early function; it is unclear why these tcfs were selected.

**Response 22:** Thank you for the question. We provided references to Tcf7 and Tcf4 (now tcf7l2) in the revised manuscript (P22 L470), and we carefully compared the maternal expression level of all TCFs, *tcf7* (previous name, *tcf1*), *tcf7l2* (previous name, *tcf4*), *tcf7l1a* (previous name, *tcf3a*) and *tcf7l1b* (previous name *tcf3b*). WISH analysis at early development stages and ovary cryo-section showed that *tcf7l2* and *tcf7* are maternally deposited (S9A and S9B Figure), which is consistent to previous report [11]. We then performed an overexpression assay, overexpression of Tcf7 and Ctnnb2 could more efficiently induce the expression of *boz* and *chd* than overexpression of Tcf7l2 and Ctnnb2 (S9C and S9D Figure), suggesting that Tcf7 could serve as a strong activator-type TCF in mediating maternal β-catenin activity in zebrafish early development, which is in accordance with previous reports showing that Tcf7 acts as β-catenin-dependent trans-activators with Lef1 [11-13]. We clarified these in the revised manuscript (P22-23 L468-484).

Reviewer #3 (Bruno Reversade, signed review):

Mudan He et al., provide a well-documented analysis of the role for Nanog in DV patterning in zebrafish using novel and genetic knockout animals. Their rescue experiments using variant Nanog constructs allowed to discriminate between the various functions of Nanog vis a vis MBT or DV axis patterning. The direct interaction and functional inhibition of maternal NANOG, via its N terminal domain towards TCF provides a compelling mechanism for the inhibition of beta-catenin-mediated WNT signaling on the ventral side.

Overall I found this manuscript to be well written, coherently assembled, scientifically sound, and with genuine mechanistic intent. As such this is an significant study which merits publication.

There are no major concerns since the author’s claims are supported by experiments which are performed with significant numbers and backed up by various orthogonal assays.
- Thank you very much for your appreciation of our work.

Major comments:

1:Based on the authors conclusion, what would be the effect of removing endogenous maternal beta-Catenin via MO depletion in MZ null null Nanog. To what extent will this rescue the phenotype. This is a more physiological assay than overexpression of gsk3b or ck1a.
**Response 23:** Thank you for the suggestion. We knocked down the maternally expressed *ctnnb2* in MZnanog embryos (presented in revised manuscript Fig 4E-4I, also see Response new Figure 4), and showed the rescue of hyper-dorsalization in MZ*nanog* (revised manuscript Fig. 4E and 4F).

2:Since nanog is a potent transcription activator,  it would be expected to have an RNAseq dataset of the transcriptome of MZ null Nanog embryos compared to wt before and after MBT to provide an unbiased profile of the differentially regulated genes. This would permit a more unbiased analysis to be done by the authors and other scientists. This should be deposited in the public domain.

**Response 24:** The original RNAseq data of the unfertilized eggs of WT and *nanog^-/-^*, and the 2 hpf, 4 hpf and 6 hpf embryos of WT and MZ*nanog* was deposited in SRA under the accession number PRJNA633216. Since the RNAseq dataset is somehow not relevant to the current study, which mainly describes a protein-protein interaction story, we prefer to present the RNAseq result in our next manuscript.

3:The authors confidently show a dorsalization of MZ null Nanog embryos but do not look at the expression of the very genes/proteins that drive ventral fates i.e., BMPs whose levels also control the expression of dorsal genes (e.g. Chd). This is lacking. The endogenous levels of expressions of Bmp2/7 and Admp by QPCR and WISH should be documented. In fact, one ligand that would be key to assess is the maternal TGFbeta Radar. Such data should be included in the revised manuscript to better describe the observed DV defects.

**Response 25:** Thank you for your suggestion. We detected the expression level of *bmp2b*, *bmp7* (S6A Fig in revised manuscript, also see Response new Figure 4), *admp* and *radar* (S6B Fig in revised manuscript, also see Response new Figure 7) in MZ*nanog* by RT-qPCR. The BMP signaling activity was reduced in MZ*nanog*, and this reduction can be rescued by knockdown of *ctnnb2*, further confirmed the dorsalized phenotype of MZ*nanog* is owing to the hyperactivation of maternal beta-catenin activity. Admp is a BMP receptor that suppresses dorsal and anterior structures and its expression is maintained by [Nodal signaling](https://www.sciencedirect.com/topics/biochemistry-genetics-and-molecular-biology/nodal-signaling) [8]. Due to the down-regulation of Nodal activity [9], the expression of *admp* is significantly reduced in MZ*nanog*. The increased expression of *radar* in MZ*nanog* at 2 hpf might be due to a negative feedback by the ectopic activation of dorsal genes. Nevertheless, the upregulated maternal *radar* in MZ*nanog* embryos and the downregulated *admp* in MZ*nanog* are fairly different from what has been described in the classical BMP-inhibited embryos [10], indicating that the dorsalization phenotype is not caused by downregulation of BMP signaling.

Response new Figure 7 Relative mRNA level of *admp* was significantly decreased and maternal radar was increased in MZ*nanog* examined by RT-qPCR analysis. *admp* was detected at 6 hpf, and *radar* was detected at 2 hpf. Error bars mean ± SD, ***P < 0.001. The P values in this figure were calculated by Student t test. The underlying data in this figure can be found in [S1 Data](https://journals.plos.org/plosbiology/article?id=10.1371/journal.pbio.3000696#pbio.3000696.s019). MZ*nanog*, maternal-zygotic mutant of nanog; RT-qPCR, reverse-transcription quantitative PCR.

4: I would like to bring to the authors’ attention that by all means zebrafish and Xenopus have very similar ways of initiating early embryogenesis both in regards to MBT, germline patterning, etc. However, there does not seem to be a Nanog homologue in Amphibians. How can they reconcile or at least discuss this conundrum?

**Response 26:** Thank you for the question. Based on sequence alignment analysis, we found no ortholog of zebrafish Nanog in Xenopus. Nevertheless, a previous study has shown that Oct25, which is also required for the maintenance of pluripotency of embryonic stem cells, exhibits a similar function in suppression of maternal β-catenin activity in Xenopus [14]. We have discussed this point in the revised manuscript (P30, L645-652).

**References**

1.	Bellipanni G. Essential and opposing roles of zebrafish  -catenins in the formation of dorsal axial structures and neurectoderm. Development. 2006;133(7):1299-309. doi: 10.1242/dev.02295.

2.	Melby AE, Beach C, Mullins M, Kimelman D. Patterning the early zebrafish by the opposing actions of bozozok and vox/vent. Developmental biology. 2000;224(2):275-85. Epub 2000/08/06. doi: 10.1006/dbio.2000.9780

S0012-1606(00)99780-X [pii]. PubMed PMID: 10926766.

3.	Kawahara A, Wilm T, Solnica-Krezel L, Dawid IB. Antagonistic role of vega1 and bozozok/dharma homeobox genes in organizer formation. Proc Natl Acad Sci U S A. 2000;97(22):12121-6. Epub 2000/10/26. doi: 10.1073/pnas.97.22.12121

97/22/12121 [pii]. PubMed PMID: 11050240.

4.	Ramel MC, Buckles GR, Baker KD, Lekven AC. WNT8 and BMP2B co-regulate non-axial mesoderm patterning during zebrafish gastrulation. Developmental biology. 2005;287(2):237-48. Epub 2005/10/12. doi: S0012-1606(05)00539-7 [pii]

10.1016/j.ydbio.2005.08.012. PubMed PMID: 16216234.

5.	Ramel MC, Lekven AC. Repression of the vertebrate organizer by Wnt8 is mediated by Vent and Vox. Development. 2004;131(16):3991-4000. Epub 2004/07/23. doi: 10.1242/dev.01277

dev.01277 [pii]. PubMed PMID: 15269175.

6.	Varga M, Maegawa S, Bellipanni G, Weinberg ES. Chordin expression, mediated by Nodal and FGF signaling, is restricted by redundant function of two beta-catenins in the zebrafish embryo. Mechanisms of development. 2007;124(9-10):775-91. Epub 2007/08/10. doi: S0925-4773(07)00101-3 [pii]

10.1016/j.mod.2007.05.005. PubMed PMID: 17686615.

7.	Perez-Camps M, Tian J, Chng SC, Sem KP, Sudhaharan T, Teh C, et al. Quantitative imaging reveals real-time Pou5f3-Nanog complexes driving dorsoventral mesendoderm patterning in zebrafish. eLife. 2016;5. doi: 10.7554/eLife.11475. PubMed PMID: 27684073; PubMed Central PMCID: PMC5042653.

8.	Lele Z, Nowak M, Hammerschmidt M. Zebrafish admp is required to restrict the size of the organizer and to promote posterior and ventral development. Dev Dyn. 2001;222(4):681-7. Epub 2001/12/19. doi: 10.1002/dvdy.1222. PubMed PMID: 11748836.

9.	Xu C, Fan ZP, Muller P, Fogley R, DiBiase A, Trompouki E, et al. Nanog-like regulates endoderm formation through the Mxtx2-Nodal pathway. Developmental cell. 2012;22(3):625-38. Epub 2012/03/17. doi: 10.1016/j.devcel.2012.01.003. PubMed PMID: 22421047; PubMed Central PMCID: PMC3319042.

10.	Yan Y, Ning G, Li L, Liu J, Yang S, Cao Y, et al. The BMP ligand Pinhead together with Admp supports the robustness of embryonic patterning. Science Advances. 2019;5(12):eaau6455. doi: 10.1126/sciadv.aau6455.

11.	Veien ES, Grierson MJ, Saund RS, Dorsky RI. Expression pattern of zebrafish tcf7 suggests unexplored domains of Wnt/beta-catenin activity. Dev Dyn. 2005;233(1):233-9. PubMed PMID: 15765502.

12.	Han W, Lee H, Han JK. Ubiquitin C-terminal hydrolase37 regulates Tcf7 DNA binding for the activation of Wnt signalling. Sci Rep. 2017;7:42590. Epub 2017/02/16. doi: 10.1038/srep42590. PubMed PMID: 28198400; PubMed Central PMCID: PMCPMC5309806.

13.	Liu F, van den Broek O, Destree O, Hoppler S. Distinct roles for Xenopus Tcf/Lef genes in mediating specific responses to Wnt/beta-catenin signalling in mesoderm development. Development. 2005;132(24):5375-85. doi: 10.1242/dev.02152. PubMed PMID: 16291789.

14.	Cao Y, Siegel D, Donow C, Knochel S, Yuan L, Knochel W. POU-V factors antagonize maternal VegT activity and beta-Catenin signaling in Xenopus embryos. Embo J. 2007;26(12):2942-54. Epub 2007/06/02. doi: 10.1038/sj.emboj.7601736. PubMed PMID: 17541407; PubMed Central PMCID: PMCPMC1894774.
