# Supplementary material for: Nanog safeguards early embryogenesis against global activation of maternal β-catenin activity by interfering with TCF factors
Source: PLoS Biol. 2020 Jul 23;18(7):e3000561. doi: 10.1371/journal.pbio.3000561 (PMC7402524; doi:10.1371/journal.pbio.3000561)
Supplement: Attachment — Submitted filename: Reviewer2_PLOS_BIOLOGY.docx [file pbio.3000561.s015.docx]

I am impressed by the amount and quality of experimental work provided by authors in response to the reviewer comments! All my concerns are fully addressed. The manuscript describes the previously unknown competition mechanism of Nanog and beta-catenin for TCF binding. The conclusions are well supported by clear experimental results, the logical flow is clear, the manuscript deserves publication. However, before publishing the manuscript, it is necessary to correct the multiple typos and language mistakes. Some (not all) of those are listed below:

Lane 68: presence (not present)

88: in (not through)

109: organizer (not organizers)

142: The nanog-deficient ES cells lost...

370: interfering with

457, 462: Word missing: These (experiments?)

466: does (not dose)

469: “To start with, we firstly identified” – reformulate this phrase

495: remove “that”

513: may interfere with

606-607: “...and there might be some negative-regulators on the coding sequence of C-terminal of Nanog” – the conclusion is unclear.
