# Supplementary material for: Nanog safeguards early embryogenesis against global activation of maternal β-catenin activity by interfering with TCF factors
Source: PLoS Biol. 2020 Jul 23;18(7):e3000561. doi: 10.1371/journal.pbio.3000561 (PMC7402524; doi:10.1371/journal.pbio.3000561)
Supplement: Attachment — Submitted filename: R2 P2P.docx [file pbio.3000561.s016.docx]

**Point-by-point response to reviewers’ comments:**

Reviewer #1: The authors have adequately addressed this reviewer's concerns by providing additional experimental data and clarifying the raised issues. The revised manuscript has be much improved. This review would recommend acceptance for publication in PLOS Biology.

-Thank you!

Reviewer #2 (Daria Onichtchouk, signed): I am impressed by the amount and quality of experimental work provided by authors in response to the requests on the first round of the review! All my concerns are fully addressed. The manuscript describes the previously unknown competition mechanism of Nanog and beta-catenin for TCF binding. The conclusions are well supported by clear experimental results, the logical flow is clear, the manuscript deserves publication.  However, before publishing the manuscript, it is necessary to correct the multiple typos and language mistakes.  Some (not all) of those are listed below:
-Thank you for your positive comments and appreciation of our revision. We have also checked the whole manuscript and corrected some typos and language mistakes.

Line 68: presence (not present)

-We have changed “present” to “presence” in revised manuscript Line 68.

88: in (not through)

-We have changed and used “in” instead of “through” in revised manuscript Line 88.

109: organizer (not organizers)

-We have changed to “organizer” in revised manuscript Line 109.

142: The nanog-deficient ES cells lost...

-We have changed “loss” to “lost” in revised manuscript Line 141.

370: interfering with

-We have added “with” after “MZnanog embryos through interfering…” in revised manuscript Line 369.

457, 462: Word missing: These (experiments?)

-Sorry for the mistakes. We have added “results” after “these” in the revised manuscript Line 455 and 460.

466: does (not dose)

-We have changed the word to “does” in revised manuscript Line 464.

469: “To start with, we firstly identified” – reformulate this phrase

-We reformulated this sentence to “Firstly, we identified” in revised manuscript Line 467.

495: remove “that”

-We have removed the word “that” in revised manuscript Line 493.

513: may interfere with

-We have added the word “with” to result in “may interfere with” in revised manuscript Line 511.

606-607: “...and there might be some negative-regulators on the coding sequence of C-terminal of Nanog” – the conclusion is unclear.

-Thank you for your pointing out this issue. We have deleted this sentence which is not related to current study.

Reviewer #3 (Bruno REVERSADE, signed): I am satisfied with the revisions.
The authors have taken at heart to respond to our concerns by direct experimentations.

-Thank you!
